# Genome-Wide Analysis of the MADS-Box Gene Family in *Brachypodium distachyon*

**DOI:** 10.1371/journal.pone.0084781

**Published:** 2014-01-13

**Authors:** Bo Wei, Rong-Zhi Zhang, Juan-Juan Guo, Dan-Mei Liu, Ai-Li Li, Ren-Chun Fan, Long Mao, Xiang-Qi Zhang

**Affiliations:** 1 State Key Laboratory of Plant Cell and Chromosome Engineering, Institute of Genetics and Developmental Biology, Chinese Academy of Sciences, Beijing, PR China; 2 National Key Facility for Crop Gene Resources and Genetic Improvement and Institute of Crop Sciences, Chinese Academy of Agricultural Sciences, Beijing, PR China; University of Connecticut, United States of America

## Abstract

MADS-box genes are important transcription factors for plant development, especially floral organogenesis. *Brachypodium distachyon* is a model for biofuel plants and temperate grasses such as wheat and barley, but a comprehensive analysis of MADS-box family proteins in *Brachypodium* is still missing. We report here a genome-wide analysis of the MADS-box gene family in *Brachypodium distachyon*. We identified 57 MADS-box genes and classified them into 32 MIKC^c^-type, 7 MIKC*-type, 9 Mα, 7 Mβ and 2 Mγ MADS-box genes according to their phylogenetic relationships to the *Arabidopsis* and rice MADS-box genes. Detailed gene structure and motif distribution were then studied. Investigation of their chromosomal localizations revealed that *Brachypodium* MADS-box genes distributed evenly across five chromosomes. In addition, five pairs of type II MADS-box genes were found on synteny blocks derived from whole genome duplication blocks. We then performed a systematic expression analysis of *Brachypodium* MADS-box genes in various tissues, particular floral organs. Further detection under salt, drought, and low-temperature conditions showed that some MADS-box genes may also be involved in abiotic stress responses, including type I genes. Comparative studies of MADS-box genes among *Brachypodium*, rice and *Arabidopsis* showed that *Brachypodium* had fewer gene duplication events. Taken together, this work provides useful data for further functional studies of MADS-box genes in *Brachypodium distachyon*.

## Introduction

MADS-box genes are important transcription factors for plant development, especially floral organ identities [1]–[2]. According to their roles in flower development, MADS-box genes are classified as having A, B, C, D and E functions [3]–[4], or are the basis for the so-called “ABCDE model” that summarizes the interactive functions of MADS-box proteins during this process [3], [5]–[7]. Generally, A- and C-lineage genes are respectively involved in sepal and carpel development. A- and B-lineage genes together are required for petal development, whereas B- and C-lineage genes are both needed for stamen development. D-lineage genes function in ovule development [5], [8]–[11], while E-lineage proteins are required for the development of all floral organs by forming MADS-box protein complexes with proteins of other lineages [11]–[12]. Evolutionarily, plant MADS-box genes are divided into type I and type II. Type II comprises most well-known MADS-box genes and can be further classified into MIKC^c^- and MIKC*-types due to differences in gene structures [13]. In general, type II proteins are composed of the most conserved MADS (M) domain for DNA binding, the keratin (K) domain for protein-protein interaction, the intervening (I) domain located between the M and K domain, and the C-terminus (C) domain that is mainly responsible for transcription activation [14]. On the other hand, MIKC*-type proteins constitute a subgroup of type II MADS-box proteins with longer I domains and less-conserved K domains when compared with MIKC^c^-type proteins [15]–[16]. Unlike type II MADS-box proteins, the structure of type I proteins is simpler which lacks the K domain. In fact, type I MADS-box genes are shared by plants and animals and thus represent a class of more ancient MADS-box genes [1], [12]. These genes are further divided into Mα, Mβ and Mγ subgroups [17]–[18]. In most plants, type I MADS-box genes experienced a faster pace of birth-and-death process than type II genes due to higher frequency of segmental gene duplications and weaker purifying selection [19].

Genome-wide analyses of MADS-box genes have been reported for *Arabidopsis*, poplar, rice, apple, cucumber, and soybean [18], [20]–[24]. Extensive work shows that, in addition to their functions in floral organ development, MADS-box genes may also be involved in abiotic responses. In wheat, for instance, *TaMADS2* was up-regulated in response to the infection of stripe rust fungus [25], while in rice, *OsMADS26*, an *AGL12* class gene, was also reported to be involved in stress tolerances [26]. Recently, flowering related MADS-box genes, such as rice *SHORT VEGETATIVE PHASE* (*SVP*)-like genes *OsMADS22* and *OsMADS55* and *Arabidopsis SUPPRESSOR OF CONSTANS 1* (*SOC1*) are found to be involved in stress tolerances [26]–[28], revealing novel roles for MADS-box genes.

*Brachypodium distachyon*, a member of the Pooideae subfamily, is a wild annual grass endemic to the Mediterranean and Middle East and belongs to the “core pooid” genera that boast the majority of important temperate cereals and forage grasses such as wheat and switchgrass [29]. It is considered a model plant with amenities of short life cycle, undemanding growing condition, and highly efficient genetic transformation system. The available of its whole genome sequence makes it a promising model for functional genomic studies of biofuel plants and temperate grasses [29], [30]. Despite these, a genome-wide investigation of *Brachypodium* MADS-box genes is still lacking. Recently, we studied the functional divergence between two duplicated D-lineage MADS-box genes, *BdMADS2* and *BdMADS4* in *Brachypodium* [31]. Here we report a more systematic study of this gene family. A total of 57 MADS-box genes were identified. A detailed phylogenetic, gene structure and conservation domain analyses were performed. In addition, we also studied the expression patterns of *Brachypodium* MADS-box genes under normal and abiotic stress conditions. We found that *Brachypodium* type I MADS-box genes are not only expressed in floral organs, but respond to abiotic stresses. Our work provides useful information on the functions of this important family of transcription factors in *Brachypodium distachyon*.

## Materials and Methods

### Plant materials

The *Brachypodium distachyon* accession BD21-3 was kindly provided by Dr. David Garvin, USDA. Plants were grown as previously described [25]. Mature root, stem, and leaf were harvested from the same plants. Flower tissues were collected at *Brachypodium* flowering time. Immature seeds were collected when the glumes were half-filled. The dissected organs were frozen immediately in liquid nitrogen and stored at -70°C till RNA extraction. For stress treatments, seeds were germinated in Hoagland solution in a petri dish on top of a Whatman 3-mm paper and vernalized at 4°C for 1–2 weeks. Plants were then transferred to a light chamber (22°C for 16 h light/18°C for 8 h dark) until emergence of the fourth leaf. For salt stress, seedlings were transferred to a fresh petri dish containing 200 mM NaCl. For cold stress, seedlings were cultured in a chamber at 4°C. To mimic drought stress, seedlings were grown in a fresh petri dish containing 18% PEG 6000. Plants were then harvested at 0, 0.5 and 1 h after abiotic treatments mentioned above. All experiments were repeated three times.

### Sequence collection and chromosome mapping

*Brachypodium* MADS-box genes were obtained by BlastP search using rice MADS-box proteins as queries against the *Brachypodium* 21-3 8X release dataset (www.brachypodium.org). ClustalX software was used to perform the multiple sequence alignment and remove redundancies [26]. Eventually, 57 MADS-box genes were identified and were named *BdMADS1-57*, including *BdMADS2* and *BdMADS4* that have been reported previously [27]. Each MADS-box gene was mapped to the *Brachypodium* genome according to their coordinates on the *Brachypodium* genome. Editseq (DNASTAR Lasergene 7.1) was employed to predict the molecular weight and isoelectric point (IP) of each protein [28].

### Phylogenetic analysis

*Brachypodium* MADS-box proteins were aligned using ClustalX with those of rice and *Arabidopsis*. An un-rooted neighbor-joining (NJ) tree was constructed using the MEGA5 package [29]. The tree nodes were evaluated by bootstrap analysis for 100 replicates [30]. Braches with less than 50% bootstrap values were collapsed. Information of all MADS-box genes including their accessions were listed in Table S2 in File S1.

### Gene structure and conserved motif analysis

Gene structures were obtained by comparisons of open reading frames (ORFs) and genomic sequences, and displayed using a gene structure display server [31]. MEME program was used to predict conserved motifs with the following parameters: number of repetitions - any, maximum number of motifs - 20, optimum motif width set to ≥ 6 and ≤ 200 [32]. The identified motifs were annotated using SMART (Simple Modular Architecture Research Tool) protein analyzing software [33]-[34].

### Calculation of Ka/Ks values and divergence times estimation

Protein sequences and ORFs of the gene pairs were aligned by DNASTAR MegAlign software, respectively. The synonymous substitution (*Ks*) and non-synonymous substitution (*Ka*) rates were calculated using the PAL2NAL web server (http://www.bork.embl.de/pal2nal/) [35], which used the CODEML program of PAML [36]. The divergence times (T) of the gene pairs were estimated using the formula: T  =  *Ks*/2λ [37], with the divergence rate λ  =  6.5×10^– 9^ [38].

### Quantitative RT-PCR

Total RNA was prepared with TRIZOL reagent following the manufacturer’s instructions (Invitrogen, CA). Reverse transcription was performed using 2.5 µg total RNA treated with RNase-free DNase I and used for first strand cDNA synthesis in a 20 µl reaction containing 10 mM DTT, 0.5 µM dNTP, 40 U RNA inhibitor, 200 U M-MLV reverse transcriptase (Promega) and 0.5 µM oligonucleotide T_15_. The reaction was performed at 42°C for 60 min with 5 min denaturation at 90°C. For gene expression quantification, specific primers were designed for each MADS-box gene (Table S3 in File S1). PCR was carried out using a Veriti 96-well Thermal Cycler (Applied Biosystems) with following programs: initial denaturation at 95°C for 3 min; 30 cycles at 95°C for 15 s, 55°C for 15 s, 72°C for 30 s, and a final extension at 72°C for 10 min or adjusted accordingly to give the best results. The expression level of *BdACT7* was used as loading control [39]. RT-PCR products were sequenced to ensure that they were derived from the desired target genes. Three independent biological replicates were performed.

## Results

### Identification and phylogenetic analysis of *Brachypodium* MADS-box genes

**Figure 1:**
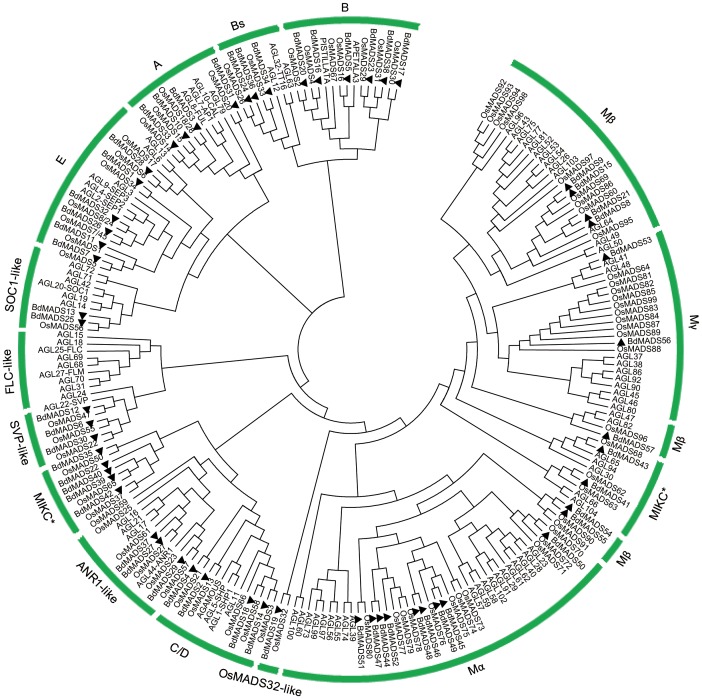
Phylogenetic analysis of MADS-box proteins in *Brachypodium*, rice and *Arabidopsis*. A total of 57 MADS-box proteins in *Brachypodium*, 75 in rice and 98 in *Arabidopsis* were used to construct the NJ tree. The MADS-box proteins in *Brachypodium* were marked by solid triangles. Branches with less than 50% bootstrap support were collapsed.

Using both type I and type II rice MADS-box domain sequences iteratively as queries to search the *Brachypodium* protein sequence dataset, a total of 57 non-redundant MADS-box proteins were identified and serially named as *BdMADS1* through *BdMADS57*, including *BdMADS2* and *BdMADS4* that have been reported before [27] (Table S1 and S2 in File S1). To determine the evolutionary relationship between *Brachypodium* MADS-box proteins and known MADS-box proteins from other species, we performed multiple sequence alignment and generated a Neighbor-Joining phylogenetic tree for MADS-box proteins from *Brachypodium*, *Arabidopsis* and rice. They were classified into functional groups according to *Arabidopsis* and rice MADS-box genes that have been extensively studied (Fig. 1) [40]. In total, 39 genes were identified as type II MADS-box genes including 32 MIKC^c^-type and 7 MIKC*-type. MIKC^c^-type MADS-box genes were divided into 9 classic clades, each of them comprised of close paralogs from rice, *Arabidopsis*, and *Brachypodium* (Fig. 1). Interestingly, *OsMADS32* and *BdMADS19* represented a novel monocot specific clade [41]. Two *Brachypodium* MADS-box genes *BdMADS41* and *BdMADS43* belong to the MIKC*-type gene group and were grouped with *Arabidopsis AGL30*, *65*, *66*, *94* and *104* and rice *OsMADS62*, *63* and *68*. The second MIKC* clade consisted *BdMADS22*, *35*, *39*, *40* and *42*, together with rice *OsMADS37*, *50* and *65*, with no *Arabidopsis* members, indicating that this group is monocot specific (Fig. 1). No *Brachypodium* and rice MADS-box gene fell in the *FLC*-clade which appeared to be *Arabidopsis* specific. On the other hand, a total of 18 MADS-box genes were identified as type I, which were further classified into Mα, Mβ and Mγ that contained 9, 7 and 2 members respectively (Fig. 1 and Table S1 in File S1). Among them, *Brachypodium BdMADS54* and *55* and rice *OsMADS90* and *91* formed a monocot-specific Mβ group.

### Gene structure and conserved motif distribution analysis

**Figure 2:**
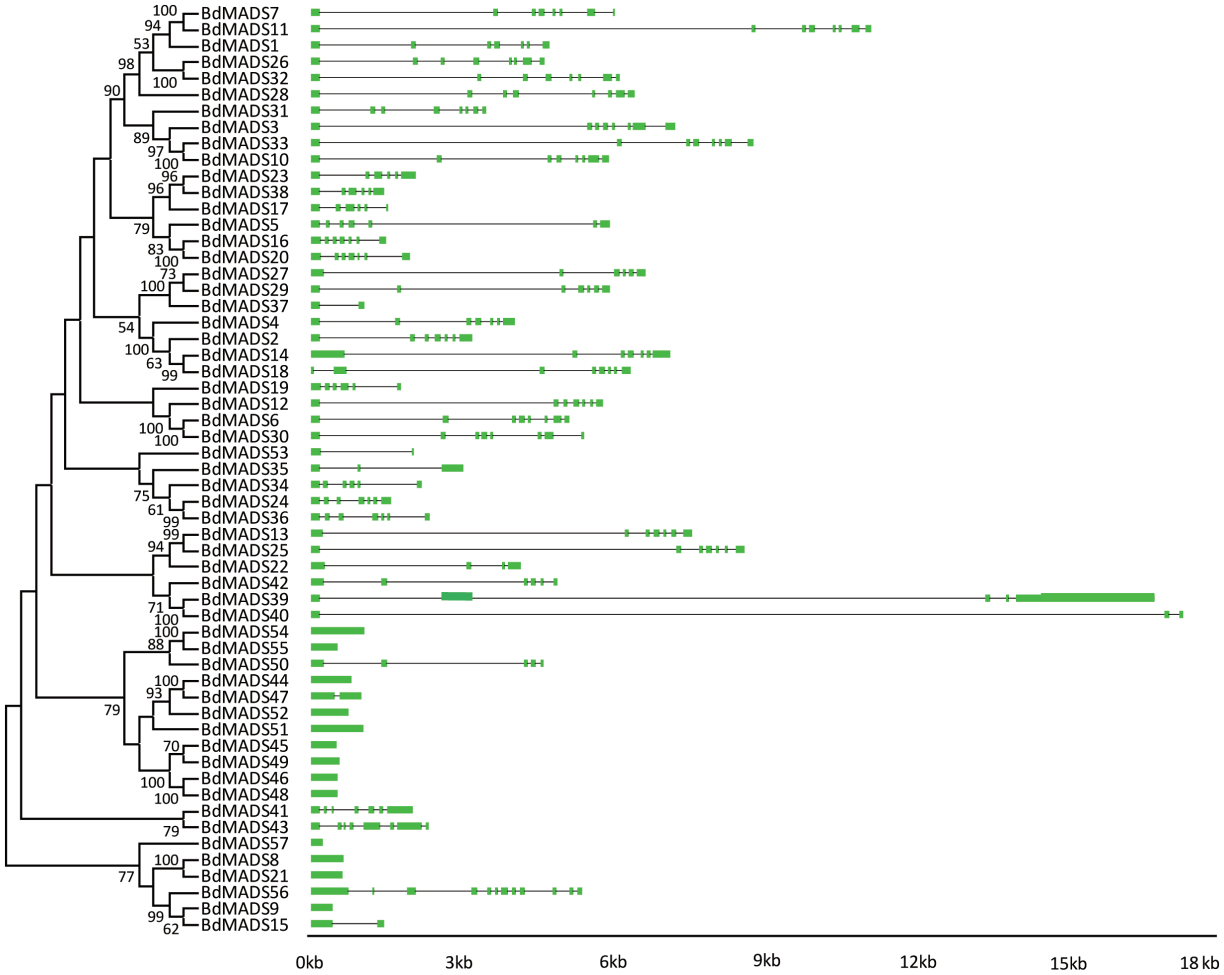
Phylogenetic relationship and gene structure analysis of MADS-box genes in *Brachypodium*. Un-rooted neighbor-joining tree was constructed from the alignment of full-length amino acid sequences using the MEGA5 package. Branches with less than 50% bootstrap values were collapsed. Lengths of exons and introns of each MADS-box gene were displayed proportionally. The green solid boxes represent exons; black lines represent introns.

**Figure 3:**
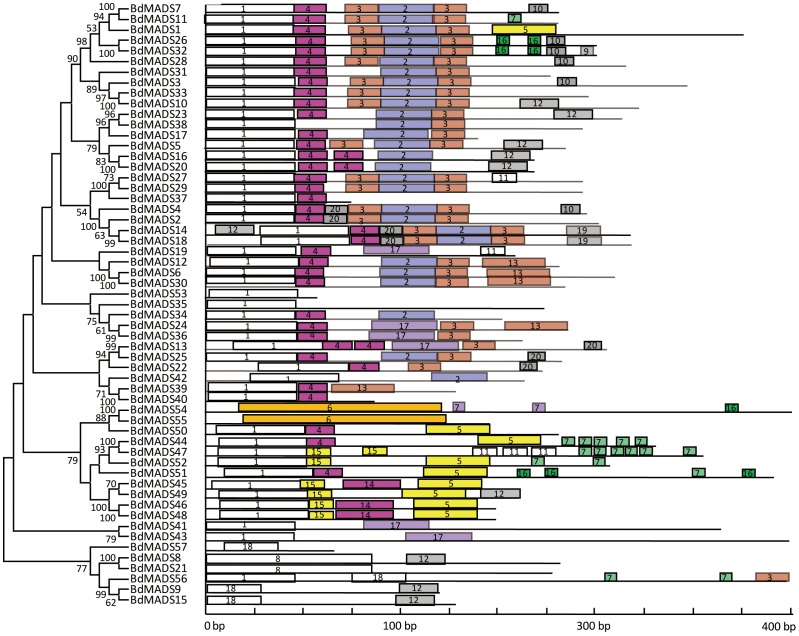
Conserved motif analysis of *Brachypodium* MADS-box proteins according to the phylogenetic relationship. Each motif is represented by a number in a colored box. Motif 1 is the MADS-box domain; 2 and 3 are two different components of the K domain; 4 and 20 are I domains; 6 is a 129 amino acid MADS-box domain; 8 is a structure; 5, 7 and 9–19 are unidentified regions. Box length corresponds to motif length. Specific lengths, locations and *p*-values of each motif can be found in Table S4 in File S1.

To better understand the structural diversity of MADS-box genes, intron/exon arrangements and conserved motifs were compared according to their phylogenetic relationships. We obtained each gene structure by comparing their ORFs with their genomic sequences. As shown in Fig. 2, closely related genes were generally more similar in gene structures, differing only in intron and exon lengths. But some close gene pairs were indeed distinct in intron/exon arrangements. For example, *BdMADS56* consisted 12 exons, whereas its close paralogues *BdMADS9* and *15* had only one and two respectively, despite that their phylogenetic relationship was supported by a 99% bootstrap value. We then used the MEME program to analyze conserved motifs in MADS-box proteins which were then subject to SMART annotation. A total of 20 conserved motifs were identified (Fig. 3 and Table S4 in File S1). Motif 1 was represented by the typical MADS-box domain of ∼57 amino acids (aa). All of type II proteins, Mα and Mγ proteins contained motif 1. The Mβ MADS-box proteins BdMADS54 and 55 harbored motif 6 which contained MADS-box domain with longer amino acids, 129 aa. In this study, motifs 2 and 3 were two fragments of the K domain, which were the second most conserved domain and essential for protein-protein interactions among MADS-box transcriptional factors [42]–[44]. Nearly all type II proteins (except for BdMDS19 and 37) contained motifs 2 and/or 3, and except for BdMADS38, all contained motif 4 or 20 that correspond to the two fragments of the I domain. For MIKC*-type proteins, BdMADS22, 39 and 40 harbored motif 4, whereas BdMADS35, 41, 42 and 43 did not. Motif 8, a coiled coil motif, was present in two recently duplicated proteins, BdMADS8 and 21. In contrast, only two type I proteins (BdMADS44 and 50) contained motif 4. Unknown motifs (motifs 5, 7, 9–19) were largely located in C-terminals, the most diverse domain of MADS-box proteins [14] and appeared mainly in recently duplicated genes. For example, nearly all Mα group proteins contained motif 5 (except for BdMADS47), suggesting that these proteins may be derived from a common ancestor.

### Genomic distribution of MADS-box genes in *Brachypodium*

**Figure 4:**
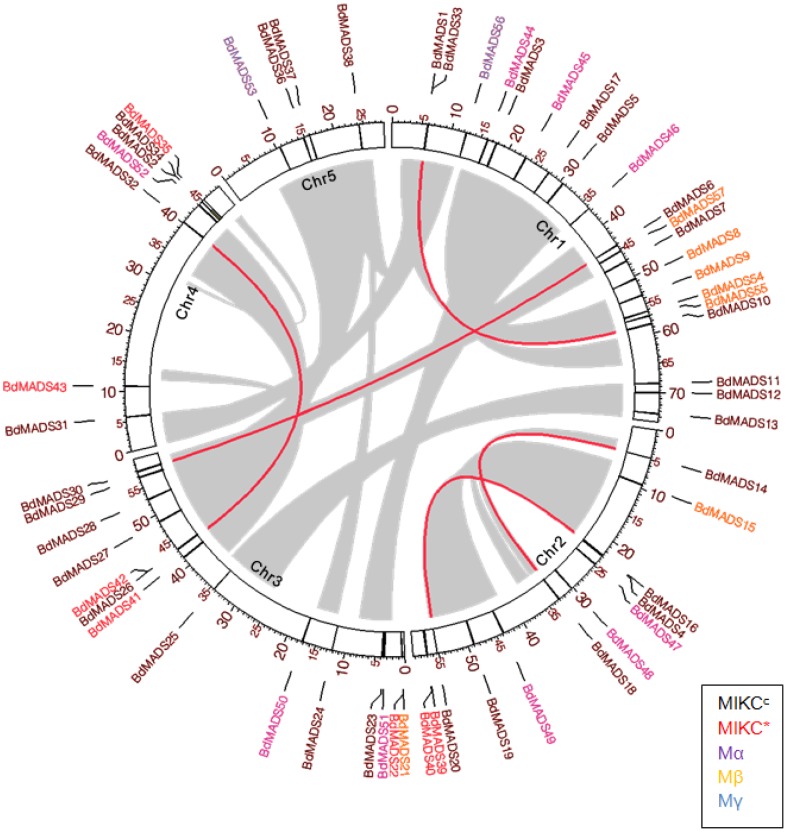
Genomic locations of MADS-box genes and duplicated gene pairs in the *Brachypodium* genome. Gene pairs located in the segmental duplicated chromosome region are linked using red lines.

To analyze genomic locations of MADS-box genes and study their evolution in the context of whole genome duplication, we mapped each gene on the *Brachypodium* genome according to their mapping coordinates. The distribution of 57 MADS-box genes on the *Brachypodium* genome appeared to be random and proportional chromosome lengths (Fig. 4). There were 20, 12, 14, 7 and 4 MADS-box genes on chromosomes 1 to 5 respectively (Fig. 4). Of these, MIKC^c^-type genes (32) were scattered across all five chromosomes, while the seven MIKC*-type genes were observed on chromosomes 2, 3 and 4. For type I genes, Mα group genes were found on chromosomes 1, 2, 3 and 4, whereas Mβ group genes were localized on chromosomes 1, 2 and 3. Two Mγ group genes were located on chromosomes 1 and 5 (Fig. 4). According to the genome duplication information and phylogenetic relationship, five pairs of type II genes were found to be located on segmental or tandem duplicated genome blocks (Fig. 4).

### Expression patterns of *Brachypodium* MADS-box genes

**Figure 5:**
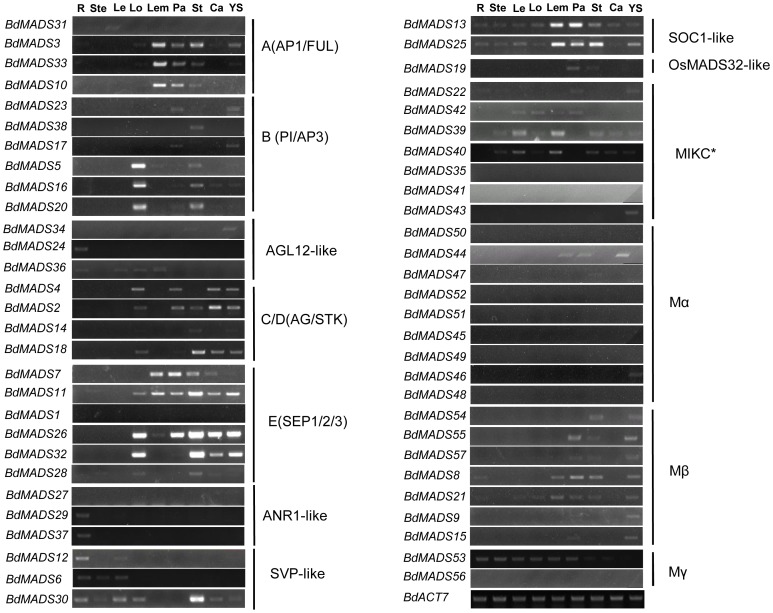
Expression patterns of *Brachypodium* MADS-box genes in vegetative and reproductive organs. Sources of the samples are as follow: root (R), stem (S), leaf (L), lodicule (Lo), lemma (Le), palea (Pa), stamen (St), carpel (Ca) and young seed (YS).

To analyze expression patterns of MADS-box genes in *Brachypodium* tissues, we harvested tissues from root, stem, leaf, and floral organs including those of lodicule, lemma, palea, stamen, carpel, and young seed. Specific primer sets were designed for each MADS-box gene and their expression patterns were detected by semi-quantitative RT-PCR (Fig. 5 and Table S3 in File S1). In general, paralogous genes of the same clade conferred similar expression patterns, although some close gene pairs did display distinct expression profiles, suggesting possible functional divergences.

**A-clade MADS-box genes.** There were four genes in the A function clade. Three of them, *BdMADS3*, *10* and *33*, were mainly expressed in reproductive organs such as lodicule, lemma, palea, stamen, and young seed. The forth one, *BdMADS31*, was only weakly expressed in leaf, a pattern similar to those of their orthologues in rice and *Arabidopsis* [18], . In addition, the weak expression signal of *BdMADS33* (but not *BdMADS10*) in young seed may indicate functional divergence of these two genes after their arise by the last genome duplication (Fig. 1, 4 and 5).

**B-clade MADS-box genes.** There were six genes in this clade. *BdMADS5* contained AP3 domain and was mainly detectable in lodicule and stamen, similar to *Arabidopsis AP3* and rice *OsMADS16*. Two *PISTILLATA* (*PI*) homologues, *BdMADS16* and *20*, were strongly expressed in lodicule and stamen and weakly expressed in palea, with *BdMADS16* additionally expressing in carpel and young seed, indicating that *BdMADS16* may also be involved in the fourth whorl development. In other words, *BdMADS16* may have expanded functions in all four floral whorls. Two other B-clade genes *BdMADS17* and *23* had low transcript levels in palea and young seed, whereas *BdMADS38* was weakly detected only in stamen. The diversified expression patterns for the B-clade MADS-box genes suggest diverged functions among these closely related genes (Fig. 5).

**C- and D-clade MADS-box genes.** This clade has four genes, with *BdMADS14* and *18* fallen into the C-lineage, whereas *BdMADS2* and *4* were of the D-lineage. *BdMADS18* was clearly expressed in stamen, carpel, young seed, lodicule, and palea, whereas *BdMADS14* was expressed preferentially in stamen and young seed. Similar expression patterns were observed for their homologs in rice [45]. For the two D-lineage MADS-box genes, as reported before [22], expression of *BdMADS2* in the carpel was much stronger than that of *BdMADS4*, indicating subfunctionalization between the two genes.

**E-clade MADS-box genes.** The E clade contains six *SEP*-like genes, including *BdMADS1*, *7*, *11*, *26*, *28* and *32*. Expressions of these were detectable in reproductive organs, however, each had a unique expression pattern. Particularly, *BdMADS11* and *BdMADS26* were expressed in all of tested reproductive tissues. In comparison, *BdMADS7* was observed in lemma, palea, stamen, and carpel. Compared to *BdMADS26*, its close paralogue *BdMADS32* was absent from the first whorl (lemma and palea), indicating functional divergence between these two genes. In addition, *BdMADS28* was weakly expressed in stamen and lodicule.

**Other MIKCc-type MADS-box genes.** Of the three *ANR1*-like clade genes, *BdMADS29* and *37* were specifically expressed in root, similar to their *Arabidopsis* homolog *ANR1* (*AGL44*) [46]. For *SVP*-clade MADS-box genes, *BdMADS6* and *BdMADS12* were preferentially expressed in root, stem, and leaf. A third *SVP* homolog *BdMADS30* was detectable not only in vegetative tissues but also in lodicule, stamen, carpel, and young seed. Both *SOC1*-clade MADS-box genes *BdMADS13* and *25* were observed to be expressed in all tested tissues. Interestingly, genes in the *AGL12*-clade displayed most diversified expression patterns. *BdMADS34* was detected in stamen and young seed, whereas *BdMADS24* was expressed specifically in root. *BdMADS36* was expressed not only in root and leaf, but also in lodicule and lemma. *BdMADS19* expression was restricted to palea and stamen, suggesting a role for development in these tissues.

MIKC*-type MADS-box genes. The five MIKC*-type genes were divergent in expression profiles. Of them, *BdMADS22* were expressed weakly in palea and young seed, whereas transcripts of *BdMADS39* and *40* were observed in nearly all organs assayed, except for root and palea (Fig. 5). Additionally, *BdMADS42* was expressed only in leaf, lodicule, lemma, and palea, while *BdMADS43* expression was observed in young seed specifically (Fig. 5).

**Type I MADS-box genes.** There are 18 type I MADS-box genes. Nine of them belong to the Mα group and only two *BdMADS44* and *BdMADS46* were found to be expressed, with *BdMADS44* expressing in palea, stamen and young seed and *BdMADS46* in young seed only (Fig. 5). In contrast, all seven genes of the Mβ group (*BdMADS8*, *9*, *15*, *21*, *54*, *55* and *57*) were expressed in young seed (Fig. 5). Additionally, *BdMADS55* and *57* also were observed in palea and stamen, whereas *BdMADS9* and *15* were expressed only in palea and *BdMADS54* was expressed only in stamen (Fig. 5). Two genes *BdMADS8* and *21* were a pair of recently duplicated genes, but with slightly different patterns. They were expressed with comparable expression patterns in root, lemma, palea, stamen, and young seed, but *BdMADS21* also expressed weakly in leaf and lodicule (Fig. 5). The Mγ group contained two genes. *BdMADS53* was strongly and preferentially expressed in root, stem, leaf, lodicule, lemma and palea, while *BdMADS56* was hardly detectable in any tissues.

In total, 12 out of the 57 *Brachypodium* MADS-box genes were found not expressed in any of the organs detected. The type I Mα group has the fewest genes (7 out of 9) expressed while the remaining groups have one or two (*BdMADS1* and *27* in MIKC^c^-type, *BdMADS35* and *BdMADS41* in MIKC*-type, and *BdMADS56* in Mγ).

### Expression analysis MADS-box genes under abiotic stress conditions

**Figure 6:**
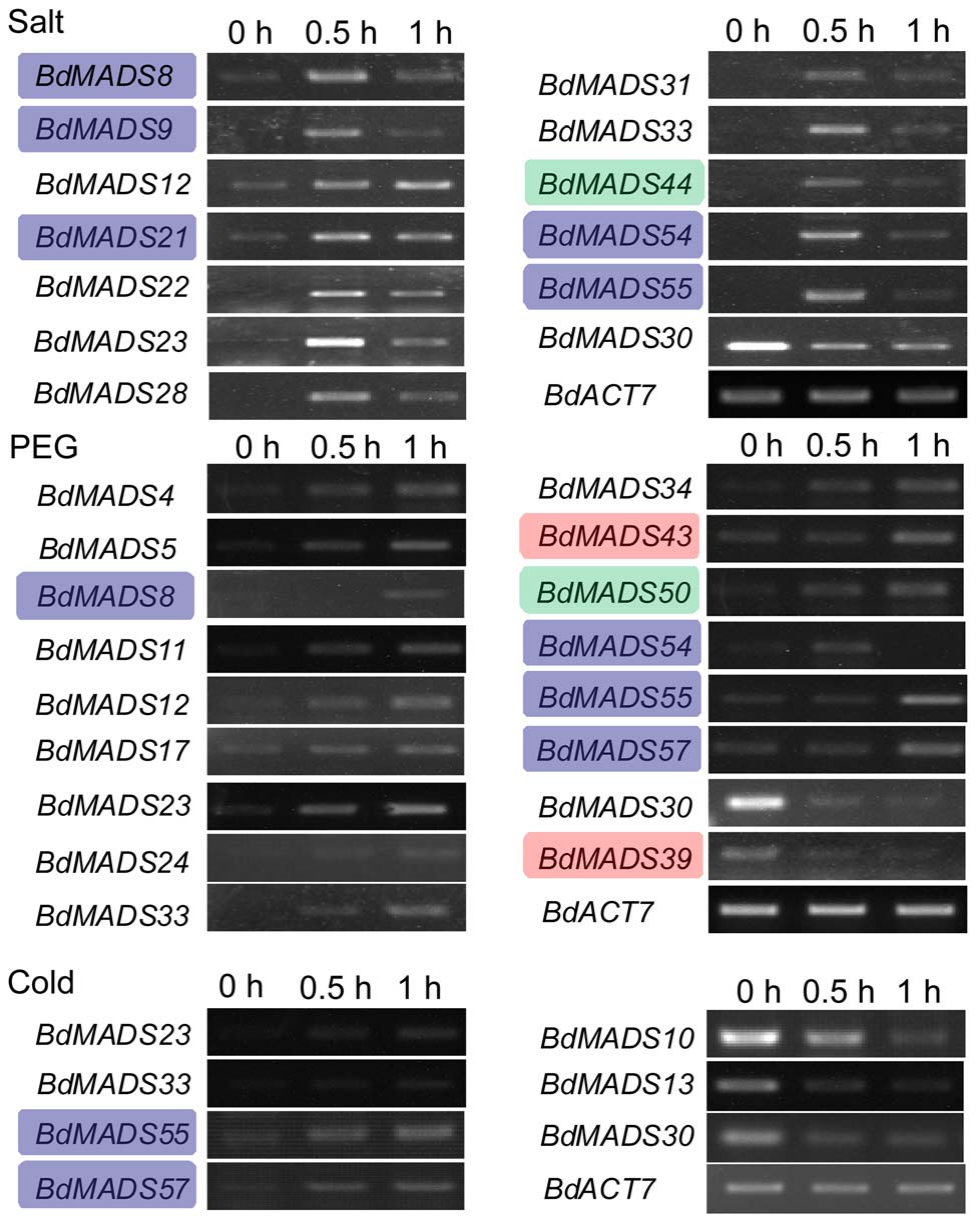
Differential expressions of MADS-box genes in response to the salt, drought, and cold stress. The Mα, Mβ and MIKC*-type genes were highlighted with green, blue and red background respectively.

The accumulative reports on MADS-box genes involving stress tolerance triggered us to investigate the responses of MADS-box genes to abiotic stresses in *Brachypodium*. As shown in Figure 6, we applied seedlings to NaCl, PEG 6000, and low-temperature treatment to mimic salt, drought, and cold stresses, respectively. The results showed that 12 MADS-box genes (*BdMADS8*, *9*, *12*, *21*–*23*, *28*, *31*, *33*, *44*, *54* and *55*) were up-regulated when seedlings were treated with 200 mM NaCl (Fig. 6) and only one gene *BdMADS30* was down-regulated. Similarly, 15 genes (*BdMADS4*, *5*, *8*, *11*, *12*, *17*, *23*, *24*, *33*, *34*, *43*, *50*, *54*, *55* and *57*) were up-regulated, and two *BdMADS30* and *BdMADS39* were down-regulated under drought stress by PEG 6000, indicating that MADS-box genes are active in response to stress conditions. The expression pattern for the cold treatment was clear but less intensive. Four genes (*BdMADS23*, *33*, *55* and *57*) were up-regulated, whereas three *BdMADS10*, *13* and *30* were down-regulated. *BdMADS30*, a *SVP*-clade gene, was also down-regulated significantly by salt and drought (Fig. 6). Thus, as suggested before, MADS-box genes in *Brachypodium* may also be involved in stress response as an escaping strategy [28].

### Duplication and functional divergence of MADS-box gene pairs in *Brachypodium*

Gene duplication events contributed significantly to the proliferation of MADS-box genes in the plant kingdom [1], [47]. Duplicated genes often evolved to partition existing functions (sub-functionalization) or obtain new functions (neo-functionalization), enhancing plants’ adaptability [48]–[49]. Using a divergence rate of 6.5×10^-9^ mutations per synonymous site per year [38], we calculated the divergence time between 18 closely related gene pairs as shown by the phylogenetic tree (Fig. 1). Table 1 shows that the divergence periods for most gene pairs were at ∼70 MYA (Million Years Ago), with a standard deviation of 20 MYA, indicating that most gene duplication events occurred before the divergence of grass species [50]. Two pairs of genes, *BdMADS39*/*40* and *BdMADS46*/*48*, were estimated to divergence at about 10 and 17 MYA and may represent two newly duplicated gene pairs, long after the *Brachypodium* diverged from other grass species. On the contrary, the divergence times of five pairs of genes (*BdMADS14*/*18*, *27*/*29*, *24*/*36*, *23*/*38* and *41*/*43*) well surpassed the divergence time of grass species (56–73 MYA) [51]–[52] and hence it is difficult to infer their actual divergence time.

**Table 1:** Estimated divergence period of MADS-box gene pairs in *Brachypodium*.

Gene pairs	*Ks*	*Ka*	*Ka*/*Ks*	MYA
*BdMADS39* vs. *BdMADS40*	0.1379	0.0001	0.001	10.6
*BdMADS46* vs. *BdMADS48*	0.224	0.0002	0.001	17.2
*BdMADS54* vs. *BdMADS55*	0.657	0.0178	0.0271	50.5
*BdMADS6* vs. *BdMADS30*	0.7259	0.0007	0.001	55.8
*BdMADS44* vs. *BdMADS47*	0.7275	0.0066	0.0091	56
*BdMADS45* vs. *BdMADS49*	0.8699	0.0085	0.0098	66.9
*BdMADS33* vs. *BdMADS10*	0.9583	0.0042	0.0044	73.7
*BdMADS16* vs. *BdMADS20*	0.9879	0.001	0.001	76
*BdMADS9* vs. *BdMADS15*	1.0037	0.001	0.001	77.2
*BdMADS13* vs. *BdMADS25*	1.0055	0.001	0.001	77.3
*BdMADS7* vs. *BdMADS11*	1.0077	0.001	0.001	77.5
*BdMADS8* vs. *BdMADS21*	1.0769	0.0011	0.001	82.8
*BdMADS32* vs. *BdMADS26*	1.1577	0.0012	0.001	89
*BdMADS14* vs. *BdMADS18*	1.5717	0.0087	0.0055	120.9
*BdMADS27* vs. *BdMADS29*	1.6545	0.0017	0.001	127.3
*BdMADS24* vs. *BdMADS36*	4.3839	0.0044	0.001	337.2
*BdMADS23* vs. *BdMADS38*	5.2382	0.0052	0.001	402.9
*BdMADS41* vs. *BdMADS43*	5.7657	0.0091	0.0016	443.5

*Ks*: synonymous substitution rate; *Ka*: non-synonymous substitution rate; MYA: million years ago.

To study the selection pressures among duplicated MADS-box genes, the substitution ratio of non-synonymous (*Ka*) to synonymous (*Ks*) mutations (*Ka*/*Ks*) were calculated for the 18 gene pairs. *Ka*/*Ks* values of all the gene pairs were less than 1, suggesting that these duplicated MADS-box gene pairs evolved under purifying selection in *Brachypodium*. In spite of this, some closely related gene pairs displayed different expression patterns indicating that have experienced subtle functional divergences. For example, among three *SVP*-like genes, *BdMADS6* and *12* were expressed dominantly in vegetative tissues, whereas *BdMADS30* was expressed in reproductive tissues, particularly in stamen, and their expression patterns were very similar to their rice homologs (Fig. 5) [53]–[54]. In addition, *BdMADS30* was down-regulated significantly by salt, drought and low-temperature stresses, however, expression of *BdMADS12* was up-regulated by salt and drought, and *BdMADS6* did not respond to abiotic stress (Fig. 6). Similarly, as two *AP1*-like genes, *BdMADS10*, but not *BdMADS33*, was down-regulated by cold treatment. Additionally, *BdMADS26* and *32* are close *SEP*-like paralogues, and their expression signals were consistently distributed in lodicule, stamen, carpel and young seed uniformly, while *BdMADS26* was also expressed in palea (Fig. 5). Expression patterns of other duplicated genes, such as *BdMADS7/11*, *BdMADS14/18*, *BdMADS46/48*, *BdMADS54/55* and *BdMADS44/47* were also diverged significantly. These data indicate that *Brachypodium* MADS-box genes are in the process of divergence under the purifying pressure by natural selection.

## Discussion

### The slow birth and death rate for *Brachypodium* MADS-box genes

Extensive studies have shown that MADS-box gene families expand by gene duplication events from whole genome duplication [52], [55]–[56]. In *Brachypodium*, there were 32 MIKC^c^-type genes, similar to those in rice (38), *Arabidopsis* (39), cucumber (29), grape (38), and poplar (55) (Table 2). *Arabidopsis* boasts the unique *FLC* clade, for which there was no counterpart in *Brachypodium* and rice. In *Arabidopsis* and *Brassicaceae*, *FLC* was identified to control flowering time and vernalization responses [57]–[59]. In temperate grass plants, *VRN1* (*BdMADS33*) was a homolog of *Arabidopsis AP1* which was up-regulated by low-temperature. However, *VRN1* responds to vernalization through a different pathway from those in *Arabidopsis* and *Brassicaceae* [60]–[61]. The fact that salt and drought stresses induced *BdMADS33* expression may suggest that *VRN1*-like genes be an important factor for abiotic stress response as well, which deserves further investigation. For *Brachypodium* type I MADS-box genes, we found that *Brachypodium* had a comparable number of Mα genes, but significantly fewer of Mβ, Mγ genes than rice and *Arabidopsis*, suggesting that rice and *Arabidopsis* genomes underwent more gene duplication events than the *Brachypodium* genome [52], [62]. In other words the *Brachypodium* genome may have experienced less gene duplication events after it diverged from rice about 40–53 MYA and hence slower birth and death rate [52].

**Table 2:** MADS-box genes in *Brachypodium*, *Arabidopsis*, rice, poplar, apple, cucumber, grapevine and soybean genomes.

Categories	*Brachypodium*	Rice	Poplar	*Arabidopsis*	Apple	Cucumber	Grapevine	Soybean
MIKCc	32	38	55	39	-	29	38	81
MIKC*	7	5	2	-	-	1	-	7
Mδ	-	-	7	6	-	3	-	-
Type II	39	43	64	54	91	33	-	88
Mα	9	13	23	25	-	5	-	37
Mβ	7	9	12	20	-	2	-	14
Mγ	2	10	6	16	-	3	-	24
Type I	18	32	41	61	56	10	-	75
Total	57	75	105	106	147	43	-	163
Reference	This study	[23]	[24]	[18]	[22]	[21]	[79]	[20]

### Differential expression of type II MADS-box genes in *Brachypodium*, rice and *Arabidopsis*

Comparing expression patterns of MADS-box gene homologous, we found that close counterparts had different expression profiles, offering clues about possible functional divergence beyond initial divergence of *Brachypodium* and rice 40–53 MYA [52]. Three *AP1*-like genes *BdMADS3*, *10* and *33* were uniformly expressed in reproductive organs, similar to what occurs in *Arabidopsis* [63]. However, rice homologues were also expressed in leaf (*OsMADS14* and *15*) and root (*OsMADS18*), besides in panicle and seed [23], [64]. Similar to expression patterns of *PI* in *Arabidopsis*, homologues in *Brachypodium* (*BdMADS16* and *20*) and rice (*OsMADS2* and *4*) also were obviously expressed in lodicule and stamen [65]–[66]. Furthermore, the subtle differences in expression patterns of each of the two *PI*-like paralogous genes in rice and *Brachypodium* likely reflected functional divergences. In addition, both *OsMADS2* and *4* were expressed in lodicule and stamen, and *OsMADS2* also was expressed in root and leaf [23]. In *Brachypodium*, *BdMADS16*, but not *BdMADS20* had weak expression signals in carpel and young seed (Fig. 5). Furthermore, *AP3* and its *Brachypodium* homolog *BdMADS5* had similar expression patterns in the second and third flower whorl, however, the rice *AP3*-like gene *OsMADS16* was expressed in the leaf and root [67]–[68]. Along with rice *OsMADS29*, *30* and *31*, *BdMADS17*, *23* and *38* formed the B-function subclade that is specific in monocot plants (Fig. 1). *OsMADS29* is interesting because it expresses specifically during S1-S4 stage of seed development, positively regulating embryo and endosperm development by affecting hormone homeostasis [23], [69]. But, no such functions were observed for the two paralogues (*OsMADS30* and *31*) [23]. The expression of the *Brachypodium* orthologue of *OsMADS29* (*BdMADS23*) can be detected in both palea and young seed, indicating that it may play a similar role, while *BdMADS38* can only be detected in stamen. These data suggest that the B-function clade genes in monocots may have significantly diversified. The *OsMADS32*-like clade was identified as a novel group specific for monocots [23], [41], [70]. In this study, *BdMADS19* transcripts were mainly detectable in palea and stamen, and this expression pattern was similar to that of *OsMADS32* [41]. However, the close homolog *TaAGL14* and *15* in wheat, was expressed in the leaf and root of most plant developmental stages other than panicle and developing seed [70]. Expression patterns of *OsMADS32*-like genes are strikingly different among monocot plants, suggesting that this young clade is undergoing dynamic functional divergence.

Functions of MIKC*-type MADS-box genes are less clearer than MIKC^c^-type genes. In *Arabidopsis*, the heterodimers of MIKC*-type proteins were required for the pollen maturation and tube growth [15]. Rice MIKC*-type genes displayed low level transcription, except for *OsMADS65* which had strong and constitutive signals [23]. In this study, MIKC*-type genes also had weak transcription signals, suggesting a need for further investigation.

### The potential functions of type I MADS-box genes

In contrast to type II MADS-box genes, not much is known about type I plant genes [71]. Recent studies suggest that type I genes are important for plant reproduction and developmental stages, especially for determining female gametophyte, embryo, and endosperm development in *Arabidopsis* [72]–[75]. As similar in *Arabidopsis*, expression of some type I genes was too weak to detect using the RT-PCR method in *Brachypodium*, and was difficult to detect using microarray techniques in rice [23], [76]–[78]. Of the nine Mα group genes, only *BdMADS44* and *46* were detectable in our studies. *BdMADS44* was mainly expressed in palea, stamen, and young seed, similar to its homologs *OsMADS77*, *78* and *79* in rice. *BdMADS46* and its rice counterpart *OsMADS75* was preferentially expressed in young seed [23]. All Mβ genes were detectable in reproductive organs. The recent duplicates *BdMADS8* and *21* were also weakly expressed in root. The expression of type I MADS-box genes in reproductive organs manifested their functions in plant reproduction. On the other hand, our work on the response of type I genes to abiotic stresses demonstrate that these ancient genes are crucial to survival of the plants under restricted growth conditions. Therefore, type I genes warrant further investigations for their roles in specifying floral organs as well as response to stressing environments.

## Supporting Information

File S1 Table S1 MADS-box genes in *Brachypodium* and their characteristics. Table S2 Accession numbers for MADS-box genes in *Brachypodium*. Table S3 Primer sets used for the semi-RT PCRs. Table S4 Conserved motifs predicted by MEME program.
(DOC)
