# Supplementary material for: Genome-Wide Analysis of the MADS-Box Gene Family in Brachypodium distachyon
Source: PLoS One. 2014 Jan 13;9(1):e84781. doi: 10.1371/journal.pone.0084781 (PMC3890268; doi:10.1371/journal.pone.0084781)
Supplement: File S1 — Table S1 MADS-box genes in Brachypodium and their characteristics. Table S2 Accession numbers for MADS-box genes in Brachypodium. Table S3 Primer sets used for the semi-RT PCRs. Table S4 Conserved motifs predicted by MEME program. (DOC) [file pone.0084781.s001.doc]

**Table S1**

**MADS-box genes in *Brachypodium* and their characteristics**.

|  | **Genes** | **Categries** | **Start** | **Stop** | **gDNA** | **cDNA** | **Exon Numbers** | **Strand** | **Length**  **(aa)** | **Molecular**  **Weight (Daltons)** | **Isoelectric**  **Point** |
| --- | --- | --- | --- | --- | --- | --- | --- | --- | --- | --- | --- |
| *BdMADS1* | *Bradi1g08330.1* | MIKCc | 5853959 | 5861589 | 5542 | 1029 | 7 | F | 342 | 38680.53 | 9.224 |
| *BdMADS2* | *Bradi4g40350.1* | MIKCc | 44750517 | 44747113 | 3405 | 756 | 7 | R | 251 | 28004.05 | 9.26 |
| *BdMADS3* | *Bradi1g21980.1* | MIKCc | 17686476 | 17678762 | 7715 | 930 | 8 | R | 309 | 35159.52 | 8.297 |
| *BdMADS4* | *Bradi2g25090.1* | MIKCc | 22913655 | 22917957 | 4303 | 732 | 7 | F | 243 | 27455.96 | 9.067 |
| *BdMADS5* | *Bradi1g35000.1* | MIKCc | 30531303 | 30524980 | 6324 | 693 | 7 | R | 230 | 26034.62 | 8.895 |
| *BdMADS6* | *Bradi1g45810.1* | MIKCc | 44095537 | 44079543 | 5875 | 780 | 8 | R | 259 | 28918.66 | 6.14 |
| *BdMADS7* | *Bradi1g48520.1* | MIKCc | 47126074 | 47132512 | 6439 | 684 | 8 | R | 227 | 26133.57 | 7.741 |
| *BdMADS8* | *Bradi1g52060.1* | Mβ | 50456290 | 50455610 | 681 | 681 | 1 | R | 226 | 24662.57 | 6.216 |
| *BdMADS9* | *Bradi1g55040.1* | Mβ | 53416340 | 53415888 | 453 | 453 | 1 | R | 150 | 15947.21 | 5.304 |
| *BdMADS10* | *Bradi1g59250.1* | MIKCc | 58361769 | 58355472 | 6298 | 837 | 8 | R | 278 | 31748.91 | 9.177 |
| *BdMADS11* | *Bradi1g69890.1* | MIKCc | 68331722 | 68343591 | 11870 | 762 | 8 | F | 253 | 29191.97 | 7.695 |
| *BdMADS12* | *Bradi1g72150.7* | MIKCc | 69952673 | 69958971 | 6299 | 675 | 7 | F | 224 | 25356.76 | 6.458 |
| *BdMADS13* | *Bradi1g77020.1* | MIKCc | 73408289 | 73416360 | 8072 | 777 | 7 | F | 258 | 29272.36 | 8.694 |
| *BdMADS14* | *Bradi2g06330.1* | MIKCc | 4764120 | 4756506 | 7615 | 810 | 7 | F | 269 | 30317.15 | 9.212 |
| *BdMADS15* | *Bradi2g11150.1* | Mβ | 9364438 | 9365981 | 1544 | 570 | 2 | F | 189 | 21177.97 | 8.788 |
| *BdMADS16* | *Bradi2g24940.1* | MIKCc | 22738068 | 22736481 | 1588 | 636 | 7 | R | 211 | 24662.26 | 8.923 |
| *BdMADS17* | *Bradi1g32210.1* | MIKCc | 27542905 | 27544536 | 1632 | 531 | 6 | F | 176 | 20261.57 | 4.771 |
| *BdMADS18* | *Bradi2g32910.1* | MIKCc | 32919230 | 32926516 | 7287 | 804 | 8 | F | 267 | 30521.42 | 8.954 |
| *BdMADS19* | *Bradi2g48690.1* | MIKCc | 48927848 | 48925857 | 1992 | 591 | 6 | R | 196 | 22381.68 | 8.402 |
| *BdMADS20* | *Bradi2g57000.1* | MIKCc | 55207867 | 55205785 | 2083 | 630 | 7 | R | 209 | 24112.65 | 7.987 |
| *BdMADS21* | *Bradi3g00720.1* | Mβ | 398641 | 397985 | 657 | 657 | 1 | R | 218 | 24282.46 | 8.732 |
| *BdMADS22* | *Bradi3g00730.1* | MIKC* | 406077 | 401649 | 4429 | 651 | 4 | R | 216 | 24252.45 | 6.118 |
| *BdMADS23* | *Bradi3g05260.1* | MIKCc | 3660927 | 3658708 | 2220 | 798 | 6 | R | 265 | 29010.79 | 6.942 |
| *BdMADS24* | *Bradi3g13570.1* | MIKCc | 12093661 | 12095344 | 1684 | 699 | 7 | F | 232 | 26121.1 | 6.458 |
| *BdMADS25* | *Bradi3g32090.1* | MIKCc | 34310788 | 34319966 | 9179(NA) | 684 | 7 | F | 227 | 25690.5 | 9.173 |
| *BdMADS26* | *Bradi3g41260.1* | MIKCc | 43213199 | 43218133 | 4935 | 753 | 8 | F | 250 | 28915.93 | 8.518 |
| *BdMADS27* | *Bradi3g46920.1* | MIKCc | 48661521 | 48668693 | 7173 | 723 | 6 | F | 240 | 27494.13 | 8.063 |
| *BdMADS28* | *Bradi3g51800.1* | MIKCc | 52807907 | 52814746 | 6840 | 807 | 8 | F | 268 | 30255.32 | 8.515 |
| *BdMADS29* | *Bradi3g57017.3* | MIKCc | 56709532 | 56703092 | 6441 | 723 | 7 | R | 240 | 27334.25 | 8.627 |
| *BdMADS30* | *Bradi3g58220.1* | MIKCc | 57745859 | 57740085 | 5775 | 690 | 8 | R | 229 | 25601.84 | 6.069 |
| *BdMADS31* | *Bradi4g06867.1* | MIKCc | 5720971 | 5724675 | 3705 | 666 | 8 | F | 221 | 24727.18 | 9.13 |
| *BdMADS32* | *Bradi4g34680.1* | MIKCc | 40216732 | 40223260 | 6529 | 744 | 8 | R | 247 | 28368.29 | 8.624 |
| *BdMADS33* | *Bradi1g08340.1* | MIKCc | 5869050 | 5878425 | 9376 | 732 | 8 | F | 243 | 27900.71 | 9.081 |
| *BdMADS34* | *Bradi4g40357.1* | MIKCc | 44757016 | 44759363 | 2348 | 567 | 6 | F | 188 | 21330.68 | 9.49 |
| *BdMADS35* | *Bradi4g40940.1* | MIKC* | 45215271 | 45212060 | 3212 | 651 | 3 | R | 216 | 24218.86 | 6.931 |
| *BdMADS36* | *Bradi5g11270.1* | MIKCc | 14827275 | 14829783 | 2509 | 609 | 7 | F | 202 | 23289.93 | 7.969 |
| *BdMADS37* | *Bradi5g12440.1* | MIKCc | 15904812 | 15905931 | 1120 | 276 | 2 | F | 91 | 10244.8 | 9.95 |
| *BdMADS38* | *Bradi5g21700.1* | MIKCc | 24247647 | 24249198 | 1552 | 723 | 6 | F | 240 | 27931.26 | 6.868 |
| *BdMADS39* | *Bradi2g59120.2* | MIKC* | 56865762 | 56881781 | 16020 | 480 | 4 | F | 159 | 17577.42 | 9.333 |
| *BdMADS40* | *Bradi2g59187.1* | MIKC* | 56959456 | 56940996 | 18461 | 324 | 3 | F | 107 | 12222.64 | 9.346 |
| *BdMADS41* | *Bradi3g39177.1* | MIKC* | 41552846 | 41550705 | 2142 | 993 | 7 | R | 330 | 36020.67 | 5.263 |
| *BdMADS42* | *Bradi3g41297.1* | MIKC* | 43258249 | 43263459 | 5211 | 612 | 6 | F | 203 | 23543.53 | 5.581 |
| *BdMADS43* | *Bradi4g11097.1* | MIKC* | 10865288 | 10862795 | 2494 | 1119 | 8 | F | 372 | 41623.95 | 6.533 |
| *BdMADS44* | *Bradi1g20090.1* | Mα | 16088153 | 16087302 | 852 | 852 | 1 | R | 283 | 30315.51 | 6.63 |
| *BdMADS45* | *Bradi1g27900.1* | Mα | 23082395 | 23082928 | 534 | 534 | 1 | F | 177 | 19548.21 | 9.415 |
| *BdMADS46* | *Bradi1g39927.1* | Mα | 36441076 | 36440516 | 561 | 561 | 1 | R | 186 | 20283.19 | 9.414 |
| *BdMADS47* | *Bradi2g26320.1* | Mα | 24742596 | 24743659 | 1064 | 945 | 2 | F | 314 | 33279.61 | 9.188 |
| *BdMADS48* | *Bradi2g30530.1* | Mα | 30210850 | 30210287 | 564 | 564 | 1 | R | 187 | 20300.35 | 9.625 |
| *BdMADS49* | *Bradi2g43290.1* | Mα | 43781813 | 43782415 | 603 | 603 | 1 | R | 200 | 21212.9 | 7.254 |
| *BdMADS50* | *Bradi3g18670.1* | Mα | 43258249 | 43263459 | 5211 | 687 | 5 | R | 228 | 25847.93 | 9.532 |
| *BdMADS51* | *Bradi3g04880.1* | Mα | 3341749 | 3342846 | 1098 | 1098 |  | F | 365 | 38245.08 | 5.756 |
| *BdMADS52* | *Bradi4g39420.1* | Mα | 44018872 | 44019648 | 777 | 777 | 1 | F | 258 | 27844.26 | 9.623 |
| *BdMADS53* | *Bradi5g08027.1* | Mγ | 10636528 | 10638691 | 2164 | 216 | 2 | F | 71 | 8189.62 | 11.086 |
| *BdMADS54* | *Bradi1g57410.1* | Mβ | 56212205 | 56213326 | 1122 | 1122 | 1 | F | 373 | 40667.57 | 4.503 |
| *BdMADS55* | *Bradi1g58100.1* | Mβ | 56957271 | 56957822 | 552 | 552 | 1 | F | 183 | 20669.35 | 6.031 |
| *BdMADS56* | *Bradi1g15480.1* | Mγ | 12416696 | 12422424 | 5729 | 1647 | 12 | F | 548 | 61185.24 | 8.408 |
| *BdMADS57* | *Bradi1g46680.1* | Mβ | 45223416 | 45223174 | 243 | 243 | 1 | R | 80 | 9394.84 | 6.866 |

***Table S2: Accession numbers for MADS-box genes in Brachypodium***

| Gene | Accession numbers ^a^ | Accession numbers (NCBI) |
| --- | --- | --- |
| *BdMADS1* | Bradi1g08330.1 | KF469299 |
| *BdMADS2* | Bradi4g40350.1 | HQ588322 |
| *BdMADS3* | Bradi1g21980.1 | KF469300 |
| *BdMADS4* | Bradi2g25090.1 | HQ588323 |
| *BdMADS5* | Bradi1g35000.1 | KF469301 |
| *BdMADS6* | Bradi1g45810.1 | KF469302 |
| *BdMADS7* | Bradi1g48520.1 | KF469303 |
| *BdMADS8* | Bradi1g52060.1 | KF469304 |
| *BdMADS9* | Bradi1g55040.1 | KF469305 |
| *BdMADS10* | Bradi1g59250.1 | HQ588324 |
| *BdMADS11* | Bradi1g69890.1 | KF469306 |
| *BdMADS12* | Bradi1g72150.7 | KF469307 |
| *BdMADS13* | Bradi1g77020.1 | KF469308 |
| *BdMADS14* | Bradi2g06330.1 | KF469309 |
| *BdMADS15* | Bradi2g11150.1 | KF469310 |
| *BdMADS16* | Bradi2g24940.1 | KF469311 |
| *BdMADS17* | Bradi1g32210.1 | KF469312 |
| *BdMADS18* | Bradi2g32910.1 | KF469313 |
| *BdMADS19* | Bradi2g48690.1 | KF469314 |
| *BdMADS20* | Bradi2g57000.1 | KF469315 |
| *BdMADS21* | Bradi3g00720.1 | KF469316 |
| *BdMADS22* | Bradi3g00730.1 | KF469317 |
| *BdMADS23* | Bradi3g05260.1 | KF469318 |
| *BdMADS24* | Bradi3g13570.1 | KF469319 |
| *BdMADS25* | Bradi3g32090.1 | KF469320 |
| *BdMADS26* | Bradi3g41260.1 | KF469321 |
| *BdMADS27* | Bradi3g46920.1 | KF469322 |
| *BdMADS28* | Bradi3g51800.1 | KF469323 |
| *BdMADS29* | Bradi3g57017.3 | KF469324 |
| *BdMADS30* | Bradi3g58220.1 | KF469325 |
| *BdMADS31* | Bradi4g06867.1 | KF469326 |
| *BdMADS32* | Bradi4g34680.1 | HQ588325 |
| *BdMADS33* | Bradi1g08340.1 | KF469327 |
| *BdMADS34* | Bradi4g40357.1 | KF469328 |
| *BdMADS35* | Bradi4g40940.1 | KF469329 |
| *BdMADS36* | Bradi5g11270.1 | KF469330 |
| *BdMADS37* | Bradi5g12440.1 | KF469331 |
| *BdMADS38* | Bradi5g21700.1 | KF469332 |
| *BdMADS39* | Bradi2g59120.2 | KF469333 |
| *BdMADS40* | Bradi2g59187.1 | KF469334 |
| *BdMADS41* | Bradi3g39177.1 | KF469335 |
| *BdMADS42* | Bradi3g41297.1 | KF469336 |
| *BdMADS43* | Bradi4g11097.1 | KF469337 |
| *BdMADS44* | Bradi1g20090.1 | KF469338 |
| *BdMADS45* | Bradi1g27900.1 | KF469339 |
| *BdMADS46* | Bradi1g39927.1 | KF469340 |
| *BdMADS47* | Bradi2g26320.1 | KF469341 |
| *BdMADS48* | Bradi2g30530.1 | KF469342 |
| *BdMADS49* | Bradi2g43290.1 | KF469343 |
| *BdMADS50* | Bradi3g18670.1 | KF469344 |
| *BdMADS51* | Bradi3g04880.1 | KF469345 |
| *BdMADS52* | Bradi4g39420.1 | KF469346 |
| *BdMADS53* | Bradi5g08027.1 | KF469347 |
| *BdMADS54* | Bradi1g57410.1 | KF469348 |
| *BdMADS55* | Bradi1g58100.1 | KF469349 |
| *BdMADS56* | Bradi1g15480.1 | KF469350 |
| *BdMADS57* | Bradi1g46680.1 | KF469351 |
| *OsMADS1* | LOC_Os03g11614 | AK070981 |
| *OsMADS2* | LOC_Os01g66030 | AK070894 |
| *OsMADS3* | LOC_Os01g10504 | AK108568 |
| *OsMADS4* | LOC_Os05g34940 | AK100233 |
| *OsMADS5* | LOC_Os06g06750 | AK064184 |
| *OsMADS6* | LOC_Os02g45770 | AK069103 |
| *OsMADS7/45* | LOC_Os08g41950 | AK100263 |
| *OsMADS8/24* | LOC_Os09g32948 | AK072867 |
| *OsMADS13* | LOC_Os12g10540 | AK070425 |
| *OsMADS14* | LOC_Os03g54160 | AK121171 |
| *OsMADS15* | LOC_Os07g01820 | AK072683 |
| *OsMADS16* | LOC_Os06g49840 | AK069317 |
| *OsMADS17* | LOC_Os04g49150 | AK070540 |
| *OsMADS18/28* | LOC_Os07g41370 | AK064704 |
| *OsMADS20* | LOC_Os12g31748 | AY250075 |
| *OsMADS21* | LOC_Os01g66290 | AK070958 |
| *OsMADS22* | LOC_Os02g52340 | AK070121 |
| *OsMADS23* | LOC_Os08g33488 | NA |
| *OsMADS25* | LOC_Os04g23910 | AK102927 |
| *OsMADS26* | LOC_Os08g02070 | AK069122 |
| *OsMADS27* | LOC_Os02g36924 | NA |
| *OsMADS29* | LOC_Os02g07430 | AK1095222 |
| *OsMADS30* | LOC_Os06g45650 | NA |
| *OsMADS31* | LOC_Os04g52410 | NA |
| *OsMADS32* | LOC_Os01g52680 | NA |
| *OsMADS33* | LOC_Os12g10520 | NA |
| *OsMADS34* | LOC_Os03g54170 | AK100227 |
| *OsMADS37* | LOC_Os08g41960 | NA |
| *OsMADS47* | LOC_Os03g08754 | NA |
| *OsMADS50* | LOC_Os03g03100 | AK104921 |
| *OsMADS55* | LOC_Os06g11330 | AK111859 |
| *OsMADS56* | LOC_Os10g39130 | AK070135 |
| *OsMADS57* | LOC_Os02g49840 | AK108784 |
| *OsMADS58* | LOC_Os05g11414 | AK111723 |
| *OsMADS59* | LOC_Os06g23950 | NA |
| *OsMADS60* | LOC_Os02g01360 | AK121824 |
| *OsMADS61* | LOC_Os04g38770 | NA |
| *OsMADS62* | LOC_Os08g38590 | NA |
| *OsMADS63* | LOC_Os06g11970 | AK111776 |
| *OsMADS64* | LOC_Os04g31804 | NA |
| *OsMADS65* | LOC_Os01g69850 | AK066160 |
| *OsMADS66* | LOC_Os05g11380 | NA |
| *OsMADS67* | LOC_Os12g31010 | ABA98556 |
| *OsMADS68* | LOC_Os11g43740 | NA |
| *OsMADS69* | LOC_Os08g20460 | NA |
| *OsMADS70* | LOC_Os05g23780 | NA |
| *OsMADS71* | LOC_Os06g22760 | NA |
| *OsMADS72* | LOC_Os03g14850 | NA |
| *OsMADS73* | LOC_Os12g21850 | NA |
| *OsMADS74* | LOC_Os12g21880 | NA |
| *OsMADS75* | LOC_Os06g30810 | NA |
| *OsMADS76* | LOC_Os06g30830 | NA |
| *OsMADS77* | LOC_Os09g02780 | NA |
| *OsMADS78* | LOC_Os09g02830 | NA |
| *OsMADS79* | LOC_Os01g74440 | NA |
| *OsMADS80* | LOC_Os02g06860 | NA |
| *OsMADS81* | LOC_Os04g24790 | NA |
| *OsMADS82* | LOC_Os04g24800 | NA |
| *OsMADS83* | LOC_Os04g24810 | NA |
| *OsMADS84* | LOC_Os04g25870 | NA |
| *OsMADS85* | LOC_Os04g25920 | NA |
| *OsMADS86* | LOC_Os03g37670 | NA |
| *OsMADS87* | LOC_Os03g38610 | NA |
| *OsMADS88* | LOC_Os01g18420 | NA |
| *OsMADS89* | LOC_Os01g18440 | NA |
| *OsMADS90* | LOC_Os07g04170 | NA |
| *OsMADS91* | LOC_Os01g11510 | NA |
| *OsMADS92* | LOC_Os01g23750 | NA |
| *OsMADS93* | LOC_Os01g23760 | NA |
| *OsMADS94* | LOC_Os01g23770 | NA |
| *OsMADS95* | LOC_Os01g23780 | NA |
| *OsMADS96* | LOC_Os01g67890 | NA |
| *OsMADS97* | LOC_Os01g68420 | NA |
| *OsMADS98* | LOC_Os01g68560 | NA |
| *OsMADS99* | LOC_Os04g25930 | NA |
| *PISTILLATA* | At5g20240 | NM_122031 |
| *APETALA3* | At3g54340 | NM_115294 |
| *AGAMOUS* | At4g18960 | NM_118013 |
| *AGL1=SHP1* | At3g58780 | NM_115740 |
| *AGL2=SEP1* | At5g15800 | NM_121585 |
| *AGL3* | At2g03710 | NM_201682 |
| *AGL4=SEP2* | At3g02310 | NM_111098 |
| *AGL5=SHP2* | At2g42830 | NM_180046 |
| *AGL6* | At2g45650 | NM_130127 |
| *AGL7=AP1* | At1g69120 | NM_105581 |
| *AGL8=FUL* | At5g60910 | NM_125484 |
| *AGL9=SEP3* | At1g24260 | NM_102272 |
| *AGL10=CAL* | At1g26310 | NM_102395 |
| *AGL11* | At4g09960 | NM_117064 |
| *AGL12* | At1g71692 | NM_105825 |
| *AGL13* | At3g61120 | NM_115976 |
| *AGL14* | At4g11880 | NM_117258 |
| *AGL15* | At5g13790 | NM_121382 |
| *AGL16* | At3g57230 | NM_115583 |
| *AGL17* | At2g22630 | NM_127828 |
| *AGL18* | At3g57390 | NM_115599 |
| *AGL19* | At4g22950 | NM_118424 |
| *AGL20=SOC1* | At2g45660 | NM_130128 |
| *AGL21* | At4g37940 | NM_119955 |
| *AGL22=SVP* | At2g22540 | NM_127820 |
| *AGL23* | At1g65360 | [NM_105210](http://www.ncbi.nlm.nih.gov/entrez/viewer.cgi?db=nucleotide&val=NM_105210) |
| *AGL24* | At4g24540 | NM_118587 |
| *AGL25=FLC* | At5g10140 | NM_121052 |
| *AGL26* | At5g26870 | BT004581 |
| *AGL27=FLM* | At1g77080 | NM_202431 |
| *AGL28* | At1g01530 | NM_105210 |
| *AGL29* | At2g34440 | NM_128996 |
| *AGL30* | At2g03060 | NM_126358 |
| *AGL31* | At5g65050 | NM_125904 |
| *AGL32=TT16* | At5g23260 | NM_122232 |
| *AGL33* | At2g26320 | [NM_128189](http://www.ncbi.nlm.nih.gov/entrez/viewer.cgi?db=nucleotide&val=NM_128189) |
| *AGL34* | At5g26575 | CW836839 |
| *AGL35* | At5g26625 | BH613122 |
| *AGL36* | At5g26645 | AY141247 |
| *AGL37* | At1g65330 | NM_105207 |
| *AGL38* | At1g65300 | NM_105204 |
| *AGL39* | At5g27130 | NM_122595 |
| *AGL40* | At4g36590 | NM_119822 |
| *AGL41* | At2g26880 | NM_128245 |
| *AGL42* | At5g62165 | NM_125610 |
| *AGL43* | At5g40220 | NM_123386 |
| *AGL44=ANR1* | At2g14210 | NM_126990 |
| *AGL45* | At3g05860 | NM_202503 |
| *AGL46* | At2g28700 | NM_128431 |
| *AGL47* | At5g55690 | NM_124951 |
| *AGL48* | At2g40210 | NM_129579 |
| *AGL49* | At1g60040 | NM_104696 |
| *AGL50* | At1g59810 | NM_104674 |
| *AGL51* | At4g02240 | AY141215 |
| *AGL52* | At4g11250 | NM_117196 |
| *AGL53* | At5g27070 | NM_122589 |
| *AGL54* | At5g27090 | NM_122591 |
| *AGL55* | At1g60920 | NM_104772 |
| *AGL56* | At1g60880 | NM_104769 |
| *AGL57* | At3g04100 | NM_111281 |
| *AGL58* | At1g28450 | NM_102613 |
| *AGL59* | At1g28460 | NM_102614 |
| *AGL60* | At1g72350 | NM_105894 |
| *AGL61* | At2g24840 | NM_179727 |
| *AGL62* | At5g60440 | NM_125437 |
| *AGL63* | At1g31140 | NM_102852 |
| *AGL64* | At1g29960 | NM_102736 |
| *AGL65* | At1g18750 | NM_101733 |
| *AGL66* | At1g77980 | NM_106447 |
| *AGL67* | At1g77950 | NM_106444 |
| *AGL68* | At5g65080 | NM_125907 |
| *AGL69* | At5g65070 | NM_125906 |
| *AGL70* | At5g65060 | NM_125905 |
| *AGL71* | At5g51870 | NM_203195 |
| *AGL72* | At5g51860 | NM_124565 |
| *AGL73* | At5g38620 | NM_123223 |
| *AGL74* | At1g48150 | NM_103711 |
| *AGL75* | At5g41200 | NM_123485 |
| *AGL76* | At5g40120 | NM_123375 |
| *AGL77* | At5g38740 | NM_123235 |
| *AGL78* | At5g65330 | NM_125931 |
| *AGL79* | At3g30260/At3g30270 | NM_113925 |
| *AGL80* | At5g48670 | NM_124244 |
| *AGL81* | At5g39750 | NM_123337 |
| *AGL82* | At5g58890 | NM_125279 |
| *AGL83* | At5g49490 | NM_124326 |
| *AGL84* | At5g49420 | NM_124319 |
| *AGL85* | At1g54760 | NM_104351 |
| *AGL86* | At1g31630 | NM_102898 |
| *AGL87* | At1g22590 | NM_202157 |
| *AGL88* | At2g11990 | AY233210 |
| *AGL89* | At5g27580 | NM_122640 |
| *AGL90* | At5g27960 | NM_122679 |
| *AGL91* | At3g66656 | NM_111544 |
| *AGL92* | At1g31640 | NM_102899 |
| *AGL93* | At5g26950 | NM_122577 |
| *AGL94* | At1g69540 | NM_105623 |
| *AGL95* | At2g15660 | NM_127127 |
| *AGL96* | At5g06500 | NM_120733 |
| *AGL97* | At1g46408 | NM_103604 |
| *AGL98* | At5g39810 | NM_123344 |
| *AGL99* | At5g04640 | NM_120546 |
| *AGL100* | At1g17310 | NM_101593 |
| *AGL101* | At5g27050 | NM_122587 |
| *AGL102* | At1g47760 | NM_103669 |
| *AGL103* | At3g18650 | NM_112751 |
| *AGL104* | At1g22130 | NM_102063 |
| *AGL105* | At5g37420 | NM_123101 |

^a^ The accession numbers of *Brachypodium* MADS-box genes are from *Brachypodium* 21-3 8 X release genes ([www.brachypodium.org](http://www.brachypodium.org/)); of rice MADS-box genes are from TIGR, of Arabidopsis MADS-box genes are from TIAR.

**Table S3**

Primer sets used for the semi-RT PCRs.

| **Gene** | **Forward primer (5'---3')** | **Reverse primer (5'---3')** |
| --- | --- | --- |
| *BdMADS1* | GTGAGGACTTGGCTCCACTTGGTA | CACGCCCTTAGATTGTGGGTTGAC |
| *BdMADS2* | AGTGCTTCTTCCCCGCCAATC | TTAGAAATGGTGAGTTTGGTCGCC |
| *BdMADS3* | AGCGTTACCAGCGGTACTCATTC | GCTTGTTGTTGCTCCATTTTGC |
| *BdMADS4* | GCTACTACCAGCACCAGCAACTCC | TTAATTGGGGCCGGTCATCTC |
| *BdMADS5* | ACAGGCAGGTGACCTACTCCAAG | GATACTTCCTCTGGCGAACCTCC |
| *BdMADS6* | CGGGAGATACGGCGGATAGAG | ATCTGCTGCAATTCACCGACACT |
| *BdMADS7* | GCAACTCCAACTCCGAAGCAAC | CCCCACATCCTGGCAAGACAT |
| *BdMADS8* | ATGCCAAAGATGAGTCAGACCG | CTGCTGCCACAAAGATAACCAC |
| *BdMADS9* | GAACTTACCCGAGAACAGTGAGCG | CACCATTGCCACGAGAACCCT |
| *BdMADS10* | GAAACTGGTGCCACGAATACA | CTGTTGAGGGACACGTGCC |
| *BdMADS11* | TACCGCACCTGCAACTACAAC | CTCCAGACTGACGGACGAAAC |
| *BdMADS12* | CGAGCCATCTCAACTGGACTT | TGGTGACGGATTCAGACGATT |
| *BdMADS13* | CAACGGCACAGCAAGACATAGA | GCAGAGGTCACCAGCGTAAGG |
| *BdMADS14* | GATCAAGCGCATCGAGAACAC | GCTCTTATCTTGGCTATGCCTTTC |
| *BdMADS15* | AGAAGGCGAGGCACGAGAATC | ATGGGTTGGAAAGACGAACTAGAAG |
| *BdMADS16* | CTGCTCGCCTAAGACCACGCTAC | GCTGCACTCGGAAGGTGAACG |
| *BdMADS17* | CGAATCCGTGGGCAGTTTGAC | GGTGCTGTGGCTGGGAAGAGT |
| *BdMADS18* | ATCAAGCGCATCGAGAACACG | CCTTTGTCCAGCCTTCCCTCC |
| *BdMADS19* | GCAGCGGCAGTCCACCTTCTA | GCCTCCGATCTTGCGGTCCTT |
| *BdMADS20* | CAAGACCTCGCTATCCAGAATC | AGCCTGTCCCAGTGCTCCATC |
| *BdMADS21* | GCAAGCGAAAGATGAACTGGA | AGGGAACGGACTGATGGAATG |
| *BdMADS22* | AGAGGAAGAGCAGCAGATCGTGG | GCGGTGGATGCGTACTCGTAG |
| *BdMADS23* | AGGGAACTCATCCAGCAGTACCAG | TCTTTCTTGCCCGGACTTTGC |
| *BdMADS24* | GCTCTATGACCTCGCCACCACC | CTGCTCATTTGCCCTGTTTCCA |
| *BdMADS25* | CGACCGCTATAAGGCATACACA | AGGGAGGGAACCACCATCAGT |
| *BdMADS26* | CCTGAAATTAAAGGCACGGGTTG | GTGGGTTCACCAGCAGCATCG |
| *BdMADS27* | GAAAGACCAAGGACGAGCAGC | TGCTCATTATCATTCTGTTGTGGC |
| *BdMADS28* | ACAGGCAACTCAAGCACAAGG | AGTGGGTTCGCAGTCCATAGC |
| *BdMADS29* | GTCGGGCTTGTCGTCTTCTC | AGTTGCTTGTGGCTTTCTTGC |
| *BdMADS30* | TCTGCACAGGGTCCTTCAAAC | TCCGAACCGTCATCATTATCC |
| *BdMADS31* | AACCTCTACACCTACTCCACCCA | CATCACCAATCTGCTGCTCAA |
| *BdMADS32* | GCGCTCATCATCTTCTCCAAC | TTCGCACAACATTTGCTCCTT |
| *BdMADS33* | CACTTGGTCGGTGATTCTGTCG | TGGTCCTGAGGTCCATGTTGTC |
| *BdMADS34* | TTGCGATGCTGAAGTTGGTGT | GCTTGTGCAATTTATCTAGCGTCAT |
| *BdMADS35* | TTGCGATGCTGAAGTTGGTGT | TTGCCCTGTCATCTTGTTCCTT |
| *BdMADS36* | ATGGCTCGTGGTAAGGTTCAG | TGTCATTGGCAGCCTTCAGTA |
| *BdMADS37* | AGGTCGGTCTTGTCATCTTCTCC | CCTTAAGCTCCGAGTTGGGGT |
| *BdMADS38* | CCGCCAAGTTACCTTCTCCAA | CAAAGTCAATGAACCCAAGTCCTC |
| *BdMADS39* | ATTTGCGGGAGCCGTAAGGAA | TCGCCAAAGCCCTCTTAGCAT |
| *BdMADS40* | CTGCTCGTCTTCTCCCCCG | CATCATTGTTGCTACTTGAATCTCC |
| *BdMADS41* | GATCGACGGCTGCGAGAAGTT | CCTGGTCACGGAGGTAGATCATG |
| *BdMADS42* | GACTCTACCCGTTCGTCTCCTC | GTTGTTCGCTTTCTCCTGTGG |
| *BdMADS43* | TTATTGGAGCGAACCTGAGAAG | CTGTGGAACCACGACGAAGAG |
| *BdMADS44* | ACCGCCAGGTCTGCTTCTCCA | GTCCTTCATCTCCATCAGTGCCG |
| *BdMADS45* | CTCGCCCTCGTCGTCTTCTCCC | CCTTGGCCTCCTCCAGCTCTGTC |
| *BdMADS46* | GCAGTGGGCGAGAAGAAGACGG | ACGTTGTACCTGAGGCGTTGGAG |
| *BdMADS47* | ACAATGCCAACACTGCTCCTGC | GCTCCCTCTTCATCTCCTCCTCC |
| *BdMADS48* | CGCAATGGGCGAGAAGAAGACG | GGCGGCGGAGTGTAGGCAAAA |
| *BdMADS49* | GAGCCCCATAGCGAACAGGAGC | CATCCCTTCCAACGCCTCACG |
| *BdMADS50* | TATCATTCATCGCTTCCTCCC | GTTTTCTTCATTTTCTTGGCTTG |
| *BdMADS51* | CCTTCGCCCACCCTTCCTTC | GTTGCACATGAGATCGGTCCCA |
| *BdMADS52* | TCGGCGGTGATTGAGGAACTGT | TGTTGGCGTTGGCGAGTAGGG |
| *BdMADS53* | CGAGCGGGGCTGATCAAGAAGG | CACTGCATGTGTGGCGGGCG |
| *BdMADS54* | GCCGTTGACTGATGAGGTGAT | GATGCAGAAGCGAAGGAGGAG |
| *BdMADS55* | ACACGGGTTGCTGTCGTCCTA | GCCACCATTCCTGGGTTCTCA |
| *BdMADS56* | TCAACCCTTTCTCGCACCCAC | TCATCTCCGCCTCCTTTCCAC |
| *BdMADS57* | AAGGTTTCCAGCAGCAACAAGT | ATCTTCTGGAGGCGAGTATCAA |
| *BdACT7* | CCTGAAGTCCTTTTCCAGCC | AGGGCAGTGATCTCCTTGCT |

**Table S4 Conserved motifs predicted by MEME program**

**Motif** 1: MADS-box

| **Name** | **Start** | ***p*-value** | **Sites** |
| --- | --- | --- | --- |
| BdMADS18 | 35 | 5.03E-67 | **MGRGRIEIKRIENTTNRQVTFCKRRNGLLKKAYELSVLCDAEVALIVFSSRGRLYEY** |
| BdMADS33 | 0 | 1.36E-66 | **MGRGRIEIKRIENTTSRQVTFCKRRNGLLKKAYELSVLCDAEVALIVFSSRGRLYEY** |
| BdMADS14 | 34 | 1.87E-66 | **MGRGRIEIKRIENTTNRQVTFCKRRNGLLKKAYELSVLCDAEVALVVFSSRGRLYEY** |
| BdMADS17 | 0 | 6.39E-66 | **MGRGKIEIKRIENTTSRQVTFCKRRNGLLKKAYELSVLCDAEIALIVFSSRGRLYEY** |
| BdMADS28 | 0 | 2.30E-64 | **MGRGRVELKRIENKINRQVTFSKRRNGLLKKAYELSVLCDAEVALIIFSSRGKLYEF** |
| BdMADS26 | 0 | 4.71E-62 | **MGRGRVELKRIENKINRQVTFAKRRNGLLKKAYELSVLCDAEVALIVFSNRGKLYEF** |
| BdMADS32 | 0 | 1.09E-61 | **MGRGRVELKRIENKINRQVTFAKRRNGLLKKAYELSVLCDAEVALIIFSNRGKLYEF** |
| BdMADS10 | 0 | 1.33E-61 | **MGRGKVQLKRIENKINRQVTFSKRRNGLLKKAHEISVLCDAEVAVVVFSPKGKLYEY** |
| BdMADS25 | 0 | 2.45E-61 | **MVRGKTELKRIENTTSRQVTFSKRRNGLLKKAFELSVLCDAEVALVVFSPRGRLYEF** |
| BdMADS7 | 0 | 5.41E-61 | **MGRGKVELKRIENKISRQVTFAKRRNGLLKKAYELSVLCDAEVALIIFSTRGRLFEF** |
| BdMADS2 | 0 | 4.39E-60 | **MGRGKVQLKRIENKINRQVTFSKRRSGLLKKAHEISVLCDAEVALIIFSTKGKLYEF** |
| BdMADS20 | 0 | 2.67E-59 | **MGRGKIEIKRIENPANRQVTFSKRRHGILKKAKEISVLCDAEVGVVIFSSAGKLYEF** |
| BdMADS3 | 0 | 1.50E-58 | **MGRGPVQLRRIENKINRQVTFSKRRNGLLKKAHEISVLCDAEVALIVFSTKGKLYEY** |
| BdMADS29 | 0 | 2.09E-58 | **MGRGKIVIRRIDNSTNRQVTFSKRRSGLLKKAKELSILCDAEVGLVVFSSTGRLYDF** |
| BdMADS11 | 0 | 4.07E-58 | **MGRGKVEMKRIENKISRQVTFAKRRNGLLKKAYELSLLCDAEVALIIFSGRGRLFEF** |
| BdMADS37 | 0 | 4.52E-57 | **MGRGKIVIRRIDNSTSRQVTFSKRRNGIFKKAKELGILCDAEVGLVIFSSTGRLYEY** |
| BdMADS27 | 0 | 1.14E-56 | **MGRGKIVIRRIDNSTSRQVTFSKRRNGIFKKAKELAILCDAEVGLMIFSSTGRLYEY** |
| BdMADS23 | 0 | 1.33E-56 | **MGRGKIEIKRIENATNRQVTFSKRRGGLLKKANELAVLCDARVGVVIFSSTGRMFEY** |
| BdMADS1 | 0 | 3.98E-55 | **MGRGKVVLQRIENKISRQVTFAKRRNGLLKKAYELSILCDAEVALVLFSHAGRLYQF** |
| BdMADS16 | 0 | 9.31E-55 | **MGRGKIEIKRIENTSNRHVTFAKRRAGLVKKAREISVLCDAEVGVVIFSSAGKLHDF** |
| BdMADS13 | 18 | 1.42E-54 | **MVRGKTQLKRIENRASRQVTFSKRRGGLRKKAHELSVLCDVEVALIVFSPSGRLYEF** |
| BdMADS42 | 28 | 7.39E-54 | **KKRGKVELRRIEDRTSRQVRFSKRRSGLFKKAFELSVLCDVEVALIVFSPAGRLYPF** |
| BdMADS40 | 1 | 4.19E-53 | **ARRGRVELRRIEDRTSRQVRFSKRRAGLFKKAFELAVLCDAEVALLVFSPAGRLYEY** |
| BdMADS31 | 0 | 5.45E-53 | **MGRGKVHLRRIENKVSRQVTFSKRRSGLLKKARELAVLCDADIAAIVFSANGNLYTY** |
| BdMADS38 | 0 | 6.22E-53 | **MGRGKVELKKIENTTSRQVTFSKRRMGLLKKANELAILCDAQIGVVIFSGSGKMYEY** |
| BdMADS24 | 0 | 1.36E-52 | **MARGKVQLRRIENPVHRQVTFCKRRAGLLKKARELSVLCDADIGIIVFSAHGKLYDL** |
| BdMADS22 | 35 | 5.52E-52 | **GRRGRREMRRIECATSRQVTFSKRRSGLLKKAFELGVLCDAEVGLLVFSPRGRLYEY** |
| BdMADS34 | 0 | 7.11E-52 | **MVRGKVRMRRIENPAHRRVTFCKRREGLLKKARELSVLCDAEVGVIIFSSQGKLHEL** |
| BdMADS35 | 0 | 7.11E-52 | **MVRGKVRMRRIENPAHRRVTFCKRREGLLKKARELSVLCDAEVGVIIFSSQGKLHEL** |
| BdMADS5 | 0 | 7.11E-52 | **MGRGKIEIKRIENATNRQVTYSKRRTGIMKKAKELTVLCDAQVAIIMFSSTGKYHEF** |
| BdMADS39 | 1 | 1.33E-51 | **VRRGRVELRRIEDRTSRQVRFSKRRSGLFKKAFELALLCDAEVALLVFSPAGKLYEY** |
| BdMADS30 | 0 | 1.21E-50 | **MARERREIKRIESSAARQVTFSKRRRGLFKKAEELSVLCDADVALIVFSSTGKLSQF** |
| BdMADS36 | 0 | 2.47E-50 | **MARGKVQMRRIENPVHRQVTFCKRRMGLLKKAKELSVLCEADIGVIVISPHGKIYEL** |
| BdMADS6 | 0 | 8.07E-50 | **MARERREIRRIESAAARQVTFSKRRRGLFKKAEELAVLCDADVALVVFSSTGKLSQF** |
| BdMADS4 | 0 | 1.02E-49 | **MGRGKVEMKRIDNDASRGVTFSKLRAGLLKKAHELAVLCDAHLGVIVFSSNGKLFDY** |
| BdMADS41 | 0 | 2.50E-48 | **MGRVKLPIKRIENNTNRHVTFSKRRNGLIKKAYELSVLCDIDIALLMFSPSKRLCPF** |
| BdMADS12 | 2 | 3.14E-47 | **GKRERIAIRRIENLAARQVTFSKRRRGLFKKAEELSILCDAEVGLAVFSATGKLFQF** |
| BdMADS19 | 0 | 4.34E-47 | **MGRGRSEIKRIENPTQRQSTFYKRRDGLFKKARELAVLCDADLLLLLFSASGKLYHY** |
| BdMADS52 | 9 | 1.74E-46 | **LGRQKIKIRRIDSDQARQVCFSKRRAGLFKKAGELSVLCGVQVAAVVFSPAGKAYSF** |
| BdMADS45 | 3 | 3.55E-45 | **RGRQRIEIRPIADTSRRQVTFSKRRSGLFKKASELCALCGADLALVVFSPAGRAFAF** |
| BdMADS47 | 8 | 1.06E-43 | **LGWQKIETKRIENQQARQVTFSKRRFGLFKKASSLSVLCGVELAAVIFSPGGKAFSF** |
| BdMADS51 | 13 | 1.44E-42 | **QGRQKIQMELIRDPNALQVCFSKRRKGLVKKVFELCVLCDAQVALVVFSPAGKPYSF** |
| BdMADS50 | 7 | 2.39E-41 | **KGRQKIEIKAIHSEKARHVCFSKRRQGLFGKANELSTLCGAGVAIVVFSPGGKIFSF** |
| BdMADS44 | 8 | 7.16E-41 | **MCRRKIAIKRIESEEDRQVCFSKRQIGLFKKVTELSVLCGMQVAVVVFSPAGNALSL** |
| BdMADS49 | 9 | 2.12E-40 | **TGRHRIEMSPIANRSSRQVTFSKRRSGFFKKGSELAILCGARVVLVVFSEVGNVFAL** |
| BdMADS46 | 12 | 3.33E-40 | **AGKRSIRIQRIENKESRLVTFSKRKSGLWKKGSEIAVLCHVRVALLAFSEAGKVFAF** |
| BdMADS43 | 0 | 3.95E-39 | **MGRVKLKIKKLENISGRHVTYSKRRSGILKKAKELSILCDIDLILLMFSPSGRPTIC** |
| BdMADS48 | 12 | 4.79E-38 | **AGKRSIRIQRIENKESRLVTFSKRKSGLWKKGSEIAVLCRVRIALLAISEAGKVFAF** |
| BdMADS53 | 2 | 4.25E-37 | **RQRGRVVLRRIEDRRRRRICFRKRRAGLIKKAEELAVLCEADVGLIVVNPFDRSFHC** |
| BdMADS56 | 0 | 2.46E-28 | **MTRKKTKIELISNASTRRSTQKKRGNGLTKKLSELLTLCGVEGCLIVFPEGDKSPPQ** |

**Motif** 2: K domain 35aa-21 to 55

| **Name** | **Start** | ***p*-value** | **Site** |
| --- | --- | --- | --- |
| BdMADS2 | 112 | 1.15E-36 | **MGEDLESLNLKELQQLEQQLESSLKHIRSRKNQLM** |
| BdMADS10 | 112 | 4.52E-34 | **MGEDLDSLNLKELQQLEQQLESSLKHIRSRKSHLM** |
| BdMADS11 | 110 | 3.70E-32 | **LGEDLGPLSMKELEQIENQIDISLKHIRSRKNQVL** |
| BdMADS3 | 113 | 5.11E-32 | **LGEQLEPLTTRELQQLEQQLDSSLKHIRSRKNQLL** |
| BdMADS7 | 110 | 2.07E-29 | **LGEDLGPLSMKELEQLENQIEISLKHIRSTKSQQS** |
| BdMADS28 | 110 | 3.46E-29 | **LGEDLGPLSVKELQQLEKQLECSLSQARQRKTQLM** |
| BdMADS32 | 114 | 1.35E-28 | **LGEDLGSLGIKELEELEKQLDSSLRHIRSTRTQHM** |
| BdMADS26 | 114 | 1.72E-28 | **LGEDLESLGIKELEGLEKQLDSSLKHIRTTRTQHM** |
| BdMADS27 | 110 | 2.70E-27 | **MGEDLSGLNVKELQSIENQLEISIRGVRTKKDQLL** |
| BdMADS38 | 109 | 1.13E-25 | **MGEDLGSLTLQDVLNLEQQIDFSLYKIRLRKQQLL** |
| BdMADS17 | 112 | 8.36E-25 | **VGESVGSLTLKELKSLENRLEKGIGRIRSKKHELL** |
| BdMADS29 | 110 | 2.50E-24 | **MGEELSGLGVTDLQGLENRLEMSLRSIKTRKDHLL** |
| BdMADS33 | 113 | 7.23E-24 | **VGDSVGNLSLKELKQLESRLEKGIAKIRARKNELL** |
| BdMADS1 | 112 | 2.03E-23 | **LGEDLAPLGTTELDQLESQVGKTLRQIRSRKTQVQ** |
| BdMADS25 | 111 | 4.33E-23 | **LGENLGECTTQELHILEAKIEKSLHIIRAKKSQLL** |
| BdMADS30 | 110 | 6.55E-23 | **RGEELDGLSVEELQQLEKKLETGLHRVLQTKDQQF** |
| BdMADS18 | 146 | 1.48E-22 | **IGESMATMSHRDLKQLEGRLDKGLGKIRARKNELL** |
| BdMADS6 | 111 | 2.84E-21 | **RGEELEGLSVGELQQMEKNLETGLQRVLCTKDQQF** |
| BdMADS14 | 146 | 5.15E-21 | **VKDSVSTMTLRDLKQLEGRLEKGIAKIRARKNELL** |
| BdMADS31 | 112 | 7.44E-21 | **MGEQLGSLTQRGVQQLEQQIGDALRSIRLRRDFLL** |
| BdMADS5 | 108 | 8.61E-21 | **MGEDLDSLEFEELRGLEQNVDAALKEVRQRKYHVI** |
| BdMADS23 | 109 | 2.73E-20 | **TGDDLSSLSLADVGDIEQQLELSASKVRARKIQLI** |
| BdMADS12 | 112 | 5.15E-20 | **RGEELQSLNIQQLQALEKRLESGLSSVLKTKSQKI** |
| BdMADS34 | 110 | 1.13E-16 | **YGGGAGDMTLDKLHKLEKGLEQWISQMRSAKMQIM** |
| BdMADS20 | 108 | 1.60E-16 | **KGEDLNSLQPKELIMIEEALDNGLTNVHEKQMEHW** |

**Motif** 3: K domain-21aa former

| **Name** | **Start** | ***p*-value** | **Site** |
| --- | --- | --- | --- |
| BdMADS33 | 92 | 3.20E-15 | **QQEAAKLRHQIQMLQNTNKHL** |
| BdMADS11 | 145 | 7.04E-15 | **LDQLFDLKNKEQELQDQNKDL** |
| BdMADS7 | 145 | 1.03E-14 | **LDQLFELKRKEQQLQDVNKDL** |
| BdMADS32 | 149 | 3.10E-14 | **LDQLTDLQRKEQMLCEANRCL** |
| BdMADS10 | 91 | 3.93E-14 | **CHEYRKLKAKIETIQKCHKHL** |
| BdMADS2 | 91 | 4.97E-14 | **CHEYRKLKAKVETIQKCQKHL** |
| BdMADS10 | 147 | 6.26E-14 | **MESISELQKKERSLQEENKAL** |
| BdMADS7 | 89 | 1.10E-13 | **YQEYLKLKTRVEFLQTTQRNL** |
| BdMADS2 | 147 | 1.53E-13 | **HESISELQRKERSLQEENKAL** |
| BdMADS18 | 181 | 1.71E-13 | **CAEIEYMQRREMELQNDNLYL** |
| BdMADS17 | 91 | 3.25E-13 | **QQESAKLRNQIQSLQSANRHL** |
| BdMADS18 | 125 | 3.61E-13 | **QQESAKLRHQITNLQNSNRTL** |
| BdMADS1 | 147 | 4.01E-13 | **LDELCDLKRKEQMLEDANLTL** |
| BdMADS32 | 93 | 6.72E-13 | **RNEYLKLKARVENLQRTQRNL** |
| BdMADS28 | 89 | 9.09E-13 | **YQEMSKLKAKLEALQRTQRHL** |
| BdMADS26 | 93 | 1.00E-12 | **RNEYLKLKARVDNLQRTQRNL** |
| BdMADS14 | 181 | 1.81E-12 | **YAEVEYMQKREMELHNDNMYL** |
| BdMADS11 | 89 | 3.87E-12 | **YQEYLKLKTRVEFLQSSQRNI** |
| BdMADS1 | 91 | 6.14E-12 | **YLEYMELKARVEVLQNSQRNL** |
| BdMADS28 | 145 | 7.37E-12 | **MEQVEELRRKERHLGEINRQL** |
| BdMADS6 | 146 | 1.15E-11 | **MQQISELQQKGTLLAEENSRL** |
| BdMADS17 | 147 | 1.50E-11 | **LAEIEYMQKMEADLQSENMYL** |
| BdMADS13 | 164 | 1.64E-11 | **EDQLAKLRQKEMTLRKENEDL** |
| BdMADS25 | 146 | 1.95E-11 | **ERQIAKLKEKETMLLKDNEEL** |
| BdMADS30 | 145 | 2.12E-11 | **LEQINELQRKSSQLAEENMQL** |
| BdMADS3 | 148 | 3.25E-11 | **FDSISELQKKEKSLKDQNGVL** |
| BdMADS26 | 149 | 3.25E-11 | **VDQLTELQRREQMFSEANKCL** |
| BdMADS27 | 145 | 4.93E-11 | **FDEIHELNRKGSMVHQENMEL** |
| BdMADS33 | 148 | 6.31E-11 | **AGEINYMAKREMELQSDNMDL** |
| BdMADS29 | 89 | 6.31E-11 | **QREAASLRQQLHNLQESHKQL** |
| BdMADS22 | 129 | 1.41E-10 | **LDQILELREKEEKLLMENSSL** |
| BdMADS31 | 147 | 1.41E-10 | **ANSIRELRNKERLLMEQNKIL** |
| BdMADS27 | 89 | 1.78E-10 | **QREAASLRQQLHNLQENHRQL** |
| BdMADS24 | 149 | 2.82E-10 | **IQEIQALKSKEDMLKAANEIL** |
| BdMADS5 | 80 | 2.82E-10 | **IEQYENMQRTLNHLKDINRNL** |
| BdMADS14 | 124 | 4.11E-10 | **QQESSKLRQQISSLQNSNSRS** |
| BdMADS29 | 145 | 6.42E-10 | **RGEIEELHRKGSLIHQENMEL** |
| BdMADS3 | 92 | 7.98E-10 | **GDEYGRLKIKLDALQKSQRQL** |
| BdMADS36 | 147 | 9.23E-10 | **SREIEMLKNKEGILKAANDML** |
| BdMADS4 | 145 | 9.23E-10 | **TEQLDETRQKVQILEDQNSFL** |
| BdMADS23 | 144 | 1.07E-09 | **NQQADNLRRKGHILEDQNTLL** |
| BdMADS12 | 147 | 1.14E-09 | **LDEISGLERKRTQLIEENSRL** |
| BdMADS16 | 141 | 6.34E-09 | **MEHWKMHRRNEKMLEDEHKLL** |
| BdMADS5 | 143 | 8.79E-09 | **TTQTETYKKKVKHSQEAYKNL** |
| BdMADS56 | 351 | 2.95E-07 | **LDTKKDLKKKEKELLAKEAEL** |

**Motif** 4: I domain-former 18aa

| **Name** | **Start** | ***p*-value** | **Site** |
| --- | --- | --- | --- |
| BdMADS10 | 58 | 6.23E-16 | **TDSSMDKILERYERYSYA** |
| BdMADS11 | 58 | 7.47E-16 | **SSSCMYKTLERYRTCNYN** |
| BdMADS2 | 58 | 4.17E-15 | **TDSCMDKILERYERYSYA** |
| BdMADS32 | 58 | 9.35E-15 | **SGQSMPKTLERYQKCSYS** |
| BdMADS26 | 58 | 1.49E-14 | **STQSMTKTLEKYQKCSYA** |
| BdMADS18 | 92 | 1.74E-14 | **SNNSVKATIERYKKATSD** |
| BdMADS7 | 58 | 2.03E-14 | **TSSCMYKTLERYRNCNSN** |
| BdMADS25 | 58 | 2.75E-14 | **SSASLQKTIDRYKAYTKD** |
| BdMADS14 | 91 | 2.75E-14 | **SNNSVKATIERYKKANSD** |
| BdMADS31 | 58 | 1.16E-13 | **TQSSMDKILERYQRCSLS** |
| BdMADS33 | 57 | 2.63E-13 | **SNNSVKATIDRYKKAHAC** |
| BdMADS27 | 57 | 2.63E-13 | **SSTSMKSVIDRYGKTKDE** |
| BdMADS30 | 57 | 3.01E-13 | **ASSSMNEIIDKYSTHSKN** |
| BdMADS6 | 57 | 3.01E-13 | **ASSSMNEIIDKYSTHSKN** |
| BdMADS1 | 58 | 5.77E-13 | **SSSNMLKTLERYQRYIYA** |
| BdMADS13 | 75 | 6.55E-13 | **ASASMQKTLERYKASTKD** |
| BdMADS37 | 57 | 1.08E-12 | **SSSSMKSVIDRYGRAKEE** |
| BdMADS17 | 57 | 1.99E-12 | **ASNSTRSTIDRYKKASAS** |
| BdMADS12 | 59 | 2.24E-12 | **ASSSMNQIIDRYNSHSKI** |
| BdMADS3 | 59 | 3.20E-12 | **QDSSMDVILERYQRYSFE** |
| BdMADS24 | 58 | 8.92E-12 | **TTGTMDGLIERYKSASGG** |
| BdMADS22 | 93 | 2.91E-11 | **STADLQKTIDRYLNHTKG** |
| BdMADS29 | 57 | 2.91E-11 | **CNTNMKAVIDRYTRAKEE** |
| BdMADS28 | 57 | 3.59E-11 | **GSAGTTKTLERYQHCCYN** |
| BdMADS40 | 59 | 3.38E-10 | **SSISIEGTYDRYQRFAGG** |
| BdMADS4 | 59 | 4.88E-10 | **PHTSWSELIQRYESSSTS** |
| BdMADS36 | 58 | 5.34E-10 | **TNGNMGSLIERYKGSNTE** |
| BdMADS39 | 59 | 1.82E-09 | **SSLSIEGTYDRYQQFAGA** |
| BdMADS50 | 64 | 2.35E-09 | **GNPSVDSIIHRFLPKSIN** |
| BdMADS44 | 65 | 3.02E-09 | **GHPSVDSVVDRLLATFTA** |
| BdMADS20 | 59 | 3.56E-09 | **SKTSLSRILEKYQINSGK** |
| BdMADS19 | 60 | 4.19E-09 | **TVPSVKEFVERYEAATQT** |
| BdMADS20 | 82 | 4.94E-09 | **KHKSLSAEIDRIKKENDN** |
| BdMADS16 | 82 | 4.94E-09 | **KHKSISAEIDRVKKENDN** |
| BdMADS16 | 59 | 6.29E-09 | **PKTTLPRILEKYQTNSGK** |
| BdMADS47 | 65 | 9.35E-09 | **GSPSVDAVINRLIATFFA** |
| BdMADS34 | 58 | 1.61E-08 | **TNGNMQSLIGRYQSDVVG** |
| BdMADS23 | 59 | 1.87E-08 | **PTSSLRELIQQYQNTTNS** |
| BdMADS51 | 70 | 3.96E-08 | **AHPSFPAVVDRFLNPQSA** |

**Motif 5: non-annotated**

| **Name** | **Start** | ***p*-value** | **Site** |
| --- | --- | --- | --- |
| BdMADS48 | 134 | 1.21E-38 | **FWFEVDVEALGAEELPVFAMALQRLRENVGRRIEFCLHSA** |
| BdMADS46 | 134 | 9.73E-38 | **FWFEVDVEALRAEELPVFAMALQRLRYNVGRRIESCLNSA** |
| BdMADS49 | 125 | 6.38E-36 | **SWWEADVEMLGEAELPEFARALKRFRDDVRRHADKLLSAP** |
| BdMADS45 | 134 | 5.01E-33 | **FYWEADVVALGEAELREFARALLRLRDDVRRRQNALLSDN** |
| BdMADS50 | 141 | 7.66E-26 | **RWLNTDVTALRLEELEEFHGELTALECMVNGRLYWLLQQA** |
| BdMADS44 | 174 | 2.16E-25 | **AWLDADLATLSESDLVEFQAALMEMKDVVQLHPDEVLREA** |
| BdMADS52 | 141 | 5.54E-23 | **LDCECELSELSEAELVDFAAALVEVQAAVQGCADERLRHA** |

**Motif** 6: MADS-box

| **Name** | **Start** | ***p*-value** | **Site** |
| --- | --- | --- | --- |
| BdMADS55 | 24 | 5.83E-147 | **FYKRHSSMFKGAADFSVLTGTRVAVVLETNGGKMHSFGTPSADPIVDAFLSENPPIGPLTDDVTS**  **ASIAWLQNEVARLDMENTPEENKTKLSIEDTKKIQRENPGMVANFIFSKQEDLSLEDLIQLFHE** |
| BdMADS54 | 21 | 7.24E-142 | **FSKRRLGLFKGASDLAAVTGARVAIVLETDSTKMHSFGTPSADPIIHAFLSGVPPPEPLTDEVMST**  **RISWLQSEVSRLDRENTSEQKRKKLVVQRIKEIQQENPGMVANHLFSKDEDLNLEDLTNLFNE** |

**Motif 7: non-annotated**

| **Name** | **Start** | ***p*-value** | **[Sites](http://ws.nbcr.net/app1334464257951/meme.html#sites_doc)** |
| --- | --- | --- | --- |
| BdMADS44 | 248 | 4.82E-11 | **MGQLPPPP** |
| BdMADS44 | 228 | 4.82E-11 | **MGQLPPPP** |
| BdMADS47 | 248 | 6.39E-11 | **MGQIPPPP** |
| BdMADS44 | 238 | 1.45E-10 | **MGHLPPPP** |
| BdMADS47 | 268 | 1.25E-08 | **TMDLPPPP** |
| BdMADS47 | 277 | 1.51E-08 | **MGQLSPFP** |
| BdMADS56 | 254 | 3.95E-08 | **TGNFPPIP** |
| BdMADS47 | 259 | 4.63E-08 | **MGQIPPFL** |
| BdMADS51 | 311 | 6.46E-08 | **MMPLPPPQ** |
| BdMADS47 | 305 | 6.46E-08 | **DGFFGPPP** |
| BdMADS44 | 274 | 6.46E-08 | **GGFFSPPP** |
| BdMADS52 | 208 | 8.85E-08 | **MMMMPPQP** |
| BdMADS47 | 237 | 1.62E-07 | **MGQIRPLP** |
| BdMADS54 | 209 | 1.62E-07 | **HGALPPAP** |
| BdMADS26 | 240 | 1.89E-07 | **MTTFMPPW** |

**Motif 8:** [**coiled coil**](http://smart.embl-heidelberg.de/smart/show_segment.pl?NAME=COIL&amp;START=18&amp;END=77&amp;BLAST=KKHAKDESDRQKQAEKKRRRLEKALANSAAIISELEKKRRQKQEEQQRLDDEGAAIAEAV)

| **Name** | **Start** | ***p*-value** | **[Sites](http://ws.nbcr.net/app1334464257951/meme.html#sites_doc)** |
| --- | --- | --- | --- |
| BdMADS8 | 0 | 8.79E-120 | **MDRAGGNQAGQVLKKGKKKHAKDESDRQKQAEKKRRRLEKALANSAAIISEL**  **EKKRRQKQEEQQRLDDEGAAIAEAVALHVLIGEDCDEPCQLMLNNRKRCDGF** |
| BdMADS21 | 0 | 2.37E-116 | **MEKAVGNQAGKVLKKGKKKQAKDELDRQKQAEKKRRRLEKALANSAAIISEL**  **EKKKQKKKEEQQRLDEEGASIAEAVALHVLIGEDSDESRHLMLNKHRRCNDW** |

**Motif 9: non-annotated**

| **Name** | **Start** | ***p*-value** | **[Sites](http://ws.nbcr.net/app1334464257951/meme.html#sites_doc)** |
| --- | --- | --- | --- |
| BdMADS16 | 182 | 4.23E-35 | **GYHHARDFAPQMPFTFRVQPSHPNLQ** |
| BdMADS20 | 180 | 2.06E-33 | **GYHTERDFAAQMPITFRVQPSHPNLQ** |

**Motif 10: non-annotated**

| **Name** | **Start** | ***p*-value** | **[Sites](http://ws.nbcr.net/app1334464257951/meme.html#sites_doc)** |
| --- | --- | --- | --- |
| BdMADS28 | 224 | 5.89E-14 | **DCEPTLQIGYP** |
| BdMADS26 | 217 | 2.66E-13 | **AGEPTLHIGYP** |
| BdMADS13 | 242 | 5.44E-11 | **DVETELFIGLP** |
| BdMADS25 | 205 | 9.97E-11 | **TVETELYIGLP** |
| BdMADS7 | 207 | 1.67E-10 | **VCDPSLHIGYQ** |
| BdMADS22 | 201 | 2.08E-10 | **DVETELVIGRP** |
| BdMADS32 | 218 | 3.67E-10 | **ASEPTLQIGFT** |
| BdMADS3 | 225 | 6.43E-10 | **DSMPNLNIGYY** |

**Motif 11: non-annotated**

| **Name** | **Start** | ***p*-value** | **[Sites](http://ws.nbcr.net/app1334464257951/meme.html#sites_doc)** |
| --- | --- | --- | --- |
| BdMADS47 | 171 | 2.70E-19 | **VYEVGGSSGGNNNGG** |
| BdMADS47 | 209 | 1.18E-18 | **VYEVGGSSVGNNNGG** |
| BdMADS47 | 190 | 2.73E-18 | **IYEVSGSSGGNNNGG** |

**Motif 12: non-annotated**

| **Name** | **Start** | ***p*-value** | **Sites** |
| --- | --- | --- | --- |
| BdMADS15 | 120 | 4.78E-30 | **ANTSASGGFSWKWHADPSASSSSF** |
| BdMADS9 | 123 | 1.18E-23 | **ANTSAGGGFSWQWCADLGGPSSSM** |
| BdMADS8 | 127 | 2.00E-18 | **ARGSHAHVPQWRWTDCGPFSFSSW** |
| BdMADS49 | 175 | 2.68E-17 | **PCTTATAAVSPGTHGDPSASDSWA** |

**Motif 13: non-annotated**

| **Name** | **Start** | ***p*-value** | **Sites** |
| --- | --- | --- | --- |
| BdMADS30 | 178 | 3.98E-44 | **LVVADTENVIAEDGQSSDSVMTALHSGSSQDNDDGSDVSL** |
| BdMADS6 | 179 | 9.58E-44 | **MTVVETENVATEDVHSSESVMTALHSGSSHDNDDGSDISL** |
| BdMADS12 | 176 | 4.17E-31 | **MQVAADSPVVYEEGQSSESVTNASYPRPPLDTEDSSDTSL** |

**Motif 14: non-annotated**

| **Name** | **Start** | ***p*-value** | **Sites** |
| --- | --- | --- | --- |
| BdMADS46 | 85 | 8.78E-46 | **PADDGAEWEAVEALYRETEGKIKEVAAESSQMDAVGEK** |
| BdMADS48 | 85 | 1.26E-45 | **PADDGAGWEAVEALYRETEGKVREVAAESARMDAVGEK** |

**Motif 15: non-annotated**

| **Name** | **Start** | ***p*-value** | **Sites** |
| --- | --- | --- | --- |
| BdMADS46 | 69 | 6.88E-16 | **GSPSVDAVLGDATG** |
| BdMADS48 | 69 | 7.91E-16 | **GSPSVDAVLGGDAG** |
| BdMADS49 | 66 | 2.39E-15 | **GSPSADAVLDGGTG** |
| BdMADS52 | 66 | 8.42E-15 | **GTPSVDAVLDRFLG** |
| BdMADS45 | 60 | 1.17E-11 | **GNPSADHVLRRHVP** |

**Motif 16: non-annotated**

| **Name** | **Start** | ***p*-value** | **Sites** |
| --- | --- | --- | --- |
| BdMADS26 | 205 | 6.80E-12 | **HGGNGFFH** |
| BdMADS32 | 206 | 6.80E-12 | **HGGNGFFH** |
| BdMADS51 | 199 | 8.08E-11 | **HGGNLFDY** |
| BdMADS51 | 217 | 4.01E-10 | **HGGDLFDY** |

**Motif 17: non-annotated**

| **Name** | **Start** | ***p*-value** | **Sites** |
| --- | --- | --- | --- |
| BdMADS24 | 105 | 9.26E-40 | **AMVLKQEIDLLQKGLRYIYGNRANEQMSVEELNSLERYLEIWMFNIRSAKMQIMIQEIQAL** |
| BdMADS36 | 103 | 1.51E-38 | **VLLLRQEIDLLQKGLRYMYGEKDINHMNLDELQALESNLEIWVHNVRSTKMQIISREIEML** |
| BdMADS13 | 120 | 7.31E-30 | **AEGLSQKLEALEAYRRKFLGEKLEDDCSFEELNSLEVKMEKSLRSIRRMKTQVFEDQLAKL** |
| BdMADS19 | 100 | 5.50E-27 | **ELENVGKMCDLLEKELRFMTVDDGEQYTVPSLAALEHNLEAAMRKVRSEKDRKIGGEMSYL** |
| BdMADS4 | 101 | 1.52E-24 | **IERLRQERDHLEASLRRLTGEDLSSLATDEELDDLEQQLQSVLGKVRQREDELLTEQLDET** |

**Motif 18: non-annotated**

| **Name** | **Start** | ***p*-value** | **Sites** |
| --- | --- | --- | --- |
| BdMADS15 | 0 | 5.68E-31 | **MDKLREQIQKARHENHKRHTASLVHNAMLGRLPGLEGLAVEEVT** |
| BdMADS9 | 0 | 5.68E-31 | **MDKLTLQIQKNLPENSERDTAILLHQAMAGRLEGGLEGLPPEKY** |
| BdMADS56 | 94 | 1.22E-30 | **MDAEGFVRELIGKLQDQLRKAERDNRERETKLLLHEVIAGRRG** |
| BdMADS57 | 11 | 5.16E-23 | **VDKEKAELFNAHRDNCDQEINLIMKQFIAHRRHS** |

**Motif 19: non-annotated**

| **Name** | **Start** | ***p*-value** | **Sites** |
| --- | --- | --- | --- |
| BdMADS18 | 231 | 2.28E-29 | **NMIHCDPRNFLQFNIMQQPQY** |
| BdMADS14 | 229 | 1.96E-24 | **HMVQYDSRNFLQVNPMQQQQQ** |

**Motif 20: I domain 17-34**

| **Name** | **Start** | ***p*-value** | **Sites** |
| --- | --- | --- | --- |
| BdMADS18 | 110 | 2.39E-20 | **TSNTGTVAEINAQHY** |
| BdMADS14 | 109 | 1.60E-19 | **TSNSGTVAEVNAQHY** |
